# Multiplicity of 5′ Cap Structures Present on Short RNAs

**DOI:** 10.1371/journal.pone.0102895

**Published:** 2014-07-31

**Authors:** Rehab F. Abdelhamid, Charles Plessy, Yoshio Yamauchi, Masato Taoka, Michiel de Hoon, Thomas R. Gingeras, Toshiaki Isobe, Piero Carninci

**Affiliations:** 1 RIKEN Center for Life Science Technologies, Division of Genomic Technologies, Yokohama, Japan; 2 RIKEN Omics Science Center, Yokohama, Kanagawa, Japan; 3 Graduate School of Science and Technology Tokyo Metropolitan University, Chemistry Department, Tokyo, Japan; 4 Core Research for Evolutional Science and Technology (CREST), Japan Science and Technology Agency (JST), Sanbancho 5, Chiyoda-ku, Tokyo, Japan; 5 Cold Spring Harbor Laboratory, Functional Genomics Laboratory, Cold Spring Harbor, New York, United States of America; University of California, Los Angeles, United States of America

## Abstract

Most RNA molecules are co- or post-transcriptionally modified to alter their chemical and functional properties to assist in their ultimate biological function. Among these modifications, the addition of 5′ cap structure has been found to regulate turnover and localization. Here we report a study of the cap structure of human short (<200 nt) RNAs (sRNAs), using sequencing of cDNA libraries prepared by enzymatic pretreatment of the sRNAs with cap sensitive-specificity, thin layer chromatographic (TLC) analyses of isolated cap structures and mass spectrometric analyses for validation of TLC analyses. Processed versions of snoRNAs and tRNAs sequences of less than 50 nt were observed in capped sRNA libraries, indicating additional processing and recapping of these annotated sRNAs biotypes. We report for the first time 2,7 dimethylguanosine in human sRNAs cap structures and surprisingly we find multiple *type 0* cap structures (mGpppC, 7mGpppG, GpppG, GpppA, and 7mGpppA) in RNA length fractions shorter than 50 nt. Finally, we find the presence of additional uncharacterized cap structures that wait determination by the creation of needed reference compounds to be used in TLC analyses. These studies suggest the existence of novel biochemical pathways leading to the processing of primary and sRNAs and the modifications of their RNA 5′ ends with a spectrum of chemical modifications.

## Introduction

The conventional and recently identified novel functional roles of RNA underscore their continuing emerging importance. Many of these roles are dependent on co- or post-transcriptional chemical modifications. Among these modifications is the addition of 7-methylguanosine cap (7 mG) to the 5′ termini of elongating mRNA transcripts. This modification has been observed to play important roles in the extra-nuclear transport, translation and overall RNA stability [1]. Beyond helping to regulate long (>200 nt) mRNAs, several biotypes of short (s) RNAs have been observed to possess 5′ cap structures [2]. sRNAs have been observed to possess a trimethylated version (2,2,7 mG) of the 7 mG cap structure. This modification has been reported as part of short nuclear RNAs (snRNAs) and nucleolar RNAs (snoRNAs). These short capped RNAs have been intensively studied for their role in gene expression, splicing regulation and long RNA editing [3]. Other modifications of 5′ caps have been found, in particular a γ-mono-methyl cap structure for the U6 snRNA [4], [5], and 2,7 mG in virus RNAs [6], [7]. In contrast, other well-characterized sRNA biotypes such as microRNAs (miRNAs), Piwi-interacting RNAs (piRNAs) and most snoRNAs, lack a 5′ cap structure, as demonstrated by the ability of cloning experiments to selectively capture these monophosphate 5′ ends without a cap [8].

While the use of a combination of 5′ cap dependent cloning (using cap analysis of gene expression, CAGE and RNA Annotation and Mapping of Promoters for the Analysis of Gene Expression, RAMPAGE) and RNA sequencing (RNAseq) has allowed for the discovery and mapping of novel long and short capped RNAs, the landscapes of modifications observed at the 5′ends of these RNAs are largely unexplored. Results from FANTOM and ENCODE projects both indicate that the total number of capped long transcripts is much greater than the protein coding RNAs in mouse [9] and human samples [10], [11]. The variety of the types of 5′ end modifications for sRNAs is particularly unexplored. A cause for this dearth of knowledge lies primarily in the reasoning that sRNAs cannot be mapped as accurately as long RNAs as well as that these sRNAs are random short-lived degradation products of long RNAs and thus are biologically unimportant.

These views have more recently come under reconsideration with the identification of novel biotypes of biologically active sRNAs. For example, sRNAs derived from processed snoRNAs [12] and tRNAs [8], [13] have been identified as being involved in gene regulation and cell proliferation, respectively. Additionally, promoter/termini-associated sRNAs (PASRs and TASRs, respectively) have been identified which when present are able to have a repressive effect on transcription [14], [15]. Notably, characterization of PASRs indicates that these sRNAs appear to be capped because these RNAs can be selectively immunoprecipated by antibodies raised against 7 mG and 2,2,7 mG cap structures [16]–[18]. Other capped sRNAs and a cytosolic capping protein complexes have been identified during studies involving the cleavage and recapping of long RNAs [14], [19]. These observations point to the possibility of not only additional capped sRNA biotypes but also to additional kinds of chemical modifications associated with the cap structures. Here, we have applied pre-[4], [20], [21] and post-genomics methods to sRNAs extracted from the THP-1 cell line to explore the range of chemical modifications found associated with the 5′cap structures.

## Materials and Methods

### Nomenclature

Methylated bases are abbreviated as follows. 1 mG: 1-methylguanosine; 2 mG: N2- methylguanosine; 7 mG: 7-methylguanosine; 2,2 mG: N2,N2-dimethylguanosine; 2,7 mG: N2,7-dimethylguanosine; 2,2,7 mG: N2,N2,7-trimethylguanosine; Cm: 2′-*O*-methylcytidine. Phosphorylated bases are indicated as follows, in combination with methylations. Np: 5′ nucleotide monophosphate. Npp: 5′ nucleotide diphosphate. Nppp: 5′ nucleotide triphosphate. NpppN: 5′-5′ nucleotide triphosphate.

### sRNA CAGE libraries

The starting material for all the libraries was sRNA isolated from THP-1 cells using the mirVana kit (Ambion) according to manufacturer’s instructions. In brief, the 3′ ends of 3 µg of sRNA were ligated to 5′ pre-adenylated and 3′ blocked adapters (5′- rApp/CTGTAGGCACCATCAAT/3ddC-3′) using a truncated T4 RNA ligase 2 enzyme (NEB). Ligated sRNAs were purified by ethanol precipitation, and reverse transcribed using an oligonucleotide complementary to the 3′ adaptor and that had an EcoP15I at its 5′ end (5′- AAGGTCTATCAGCAGAAAAATTGATGGTGCCTACAG-3′). The later steps of libraries preparation were performed according the CAGE protocol [22]. The cDNAs were amplified using 18 PCR cycles using a primers targeting the 5′ linker and 3-linker with additional sequence for bridge PCR on Illumina sequencers.

### Immunoprecipitated sRNAs

Three batches of 2×10^7^ THP-1 cells were lysed using the mirVana Kit lysis buffer (600 µl, Life Technologies). The cell lysate was diluted in 10×IP buffer (25 mM Tris-HCl pH 7.5, 150 mM KCl, 1 mM DTT, 1×protease inhibitor, 0.1% NP40). The mixture was centrifuged 14,000 rpm for 15 min at 4°C. The supernatants were divided into three tubes for immunoprecipitation with the monoclonal K121 antibody, and three tubes for the IP negative controls. 10 µg of antibody or 1 ml of RNase-DNase free water (Gibco) for the negative controls were mixed with 1 ml of cell lysate in the presence of 40 U of RNaseOUT and rotated overnight at 4°C. 100 µl of washed Dynabeads protein G (cat. #100.03 Invitrogen) were added and the tubes were rotated at 4°C for an extra 5 h. G beads were washed 3×10 min at 4°C with 200 µl IP buffer, re-suspended in 200 µl IP buffer and then supplemented with 50 µg Protease K. RNA was separated from the beads by incubating at 40°C for 30 min, extracted with phenol/chloroform and ethanol precipitated.

### Capped sRNA libraries

We prepared libraries from the immunoprecipitated RNA and from 1.6 µg aliquots of THP-1 sRNA extracted by the mirVana kit (Ambion). All RNAs except for one set of control libraries (to capture sRNAs that have a 5′ phosphate) were dephosphorylated with shrimp alkaline phosphatase (SAP, USB) according to the manufacturer’s recommendation, and then decapped with 1 U of tobacco acid pyrophosphatase (TAP, Epicentre) in the presence of 20 U of RNaseOUT. The moderate amount of TAP was chosen because of a residual RNAse activity of the enzyme (Figure S1 in File S1). The decapped RNA was recovered by extraction with phenol/chloroform and ethanol precipitated, and used to prepare libraries based on 5′ adapter ligation [23].

### Sequencing and data analysis

Libraries were loaded in a HiSeq 2000 sequencer (Illumina) at a 10 pM final concentration, with single reads of 100 nt. All the data has been deposited in the DNA DataBank of Japan’s Sequence Read Archive (DDBJ SRA) under the accession number DRA001138.

The reads contained sample indexes used by the HiSeq software pipeline to de-multiplex the libraries. Tags were clipped from the reads using the FASTX-Toolkit program (http://hannonlab.cshl.edu/fastx_toolkit/), requiring a match of at least 8 bases. Thus, we could infer the length of the RNAs shorter than 93 nt. 100-nt tags represent longer RNAs. Unlike in other libraries, the *sRNA CAGE* tags had a fixed size peaking at 27 nt because of the enzymatic cleavage by the EcoP15I enzyme. We removed all the tags that had exact matches with the reference human ribosomal DNA unit (GenBank: U13369) using the rRNAdust program (available upon request). The reads were then aligned with BWA [24].

The TSS and TTS plots were calculated by counting the number of reads aligned to windows of 100 nt centered on the start and end positions from the “protein_coding” or “lncRNA” transcript models of GENCODE 17. Counts per category were computed with BEDTools [25] and R (R Foundation for Statistical Computing, 2008, ISBN 3-900051-07-0) with scripts available upon request.

We annotated the aligned sRNAs using our in-house annotation pipeline (source code available upon request). The annotations were then resolved hierarchically with the following categories in increasing order of priority: unknown, intron, exon, miRNA, snRNA, snoRNA, tRNA.

### RNA fractionation by electrophoresis for thin layer chromatography (TLC)

sRNAs were isolated from THP-1 cells using the mirVana kit (Ambion) according to the manufacturer’s instructions, precipitated with isopropanol and re-suspended in 10 µl RNase-DNase free water (Gibco), mixed with equal volume of gel loading dye (Ambion), heat denatured for 5 min and then cooled immediately on ice before separation on a 15% (w/v) denaturing acrylamide gel (1×TBE, 7 M urea, 15% acrylamide (29∶1 acryl:bis-acryl) in 1×TBE running buffer. The gel was stained with SYBR Gold (Invitrogen, Carlsbad, CA) for 5 min, and size fractions were collected by cutting the gel into three slices (<50 nt, 50–100 nt, 100–200 nt) according to a RNA molecular weight marker (RNA lowII, DynaMarker) (Figure S2 in File S1). The gel slices were transferred to a 2 ml clean tube RNase- and DNase-free and smashed by passing through a membrane-less filter (illustra MicroSpin columns, GE Healthcare). RNA was eluted in 1 ml of TEN buffer (Tris-HCl (pH 8.0) 8 mM, EDTA 0.1 M, NaCl 240 mM in RNase-free water), rocking overnight at 4°C. The tubes were then centrifuged 5 min at 10,000×*g* speed, the aqueous phase was recovered and the RNA was ethanol precipitated and re-suspended in RNase, DNase free water.

### Preparing the 5′ cap standards

For the preparation of cap standards, a T7 RNA polymerase promoter was incorporated into a PCR product containing 326 bp of the bacteriophage lambda genome (GenBank: J02459.1, Lambda Phage DNA: Wako cat. #91080-14-7) with the forward primer TAATACGACTCACTATAGGGcgtttccgttcttcttcgtc and the reverse primer cgcagcttttcgttctcaat. The gel extracted PCR product was used as template for T7 RNA transcription (MEGAscript T7, Ambion). The purified RNA fragment was capped using vaccinia virus capping enzyme (Ambion) in the presence of [α-^32^P]GTP. The ^32^P labeled 7 mG cap was purified to partially remove unincorporated [α-^32^P]rGTP by three consecutive ProbeQuant G-50 micro column (GE healthcare), using the remaining unincorporated Gppp (further shortened to Gp in a later step) as one of the standards. Methyltransferase reactions (10 µl) were performed according to literature [26] using the trimethylguanosine synthase from *Giardia lamblia* (GlTgs2) to prepare 2,7 mG-capped RNA, and human TGS-1 (huTgs1) to prepare 2,2,7 mG-capped RNA. GITgs2 (GenBank accession number XP_001704513) cloned into the *Escherichia coli* expression vector pET200D (Invitrogen) plasmid was a generous gift from Dr Barbosa at the University of California, Los Angeles. Induction of expression and purification of the N-terminal His-tagged GlTgs2 was carried out as described [27]. Short isoform of TGS-1 (GST-huTgs1-sf) plasmid (pGEX background) was a generous gift from Dr Bordonne at Université Toulouse III Paul Sabatier. Induction of expression and purification of the GST-tagged huTgs1-sf were carried out as described in [28]. Protein purity and concentration were evaluated by SDS-PAGE and densitometry using BSA standards. The labeled cap structures were released with TAP (Epicentre), using the same digestion procedure as above.

### Determination of the affinity of the K121 antibody to methylguanosines

Standard capped RNAs were mixed in IP buffer (25 mM Tris-HCl pH 7.5, 10 mM MgCl_2_, 0.5 mM DTT, 1×protease inhibitor). The mixture was divided into four aliquots of 200 µl, three for the immunoprecipitation, and the fourth for IP library negative control. 30 µg (300 µl) of mouse monoclonal antibody against 7 mG and 2,2,7 mG caps clone K121 (Calibochem cat. #D00036157) [29] and 300 µl of RNase- DNase free water (Gibco) for the negative control were mixed with standard capped RNAs in the presence of 40 U of RNaseOUT and rotated over night at 4°C. 100 µl of washed Dynabeads protein G (cat. #100.03 Invitrogen) were added to each tube and the tubes were rotated at 4°C for an extra 5 h. G beads were washed 3×10 min at 4°C with 200 µl IP buffer, re-suspended in 200 µl IP buffer and then supplemented with 50 µg Protease K. The RNAs were separated from the beads by incubating at 40°C for 30 min, extracted with phenol/chloroform and ethanol precipitated. The labeled cap structures were released with tobacco acid pyrophosphatase (TAP, Epicentre), using the manufacturer’s procedure. The replicates were analysis on one or two dimension TLC system as described below (Figures S3–S5 in File S1).

### 5′ end labeling of fractionated RNA

Fractionated RNA was decapped 1 h at 37°C in a volume of 10 µl containing 50 mM sodium acetate pH 5.1, 1 mM EDTA, 20 U RNaseOUT (Invitrogen), and 1 U TAP (Epicenter)/150 pmol RNA. The enzyme was heat-inactivated at 65°C for 10 min, and the cleavage products were dephosphorylated by adding 1 mM MgCl_2_ and 5 U of antarctic phosphatase (AP, NEB), incubating at 37°C for 30 min then heat inactivating at 65°C for 20 min. The 5′ cap structures were radiolabelled with 4 U T4 polynucleotide kinase (PNK, USB) and 2 mM final concentration of ATP, in 1×PNK buffer in the presence of 50 pmol [γ-^32^P]ATP (3000 Ci/mmol) [30]. T4 PNK reaction was incubated at 37°C for 1 h and then deactivated at 65°C for 10 min. 1 µl of apyrase (NEB) was added to the reaction mixture to digest the un-incorporated [γ-^32^P]ATP.

Aliquots of individual RNA fraction’s reactions were analyzed by 2D TLC analysis with 10×10 cm plain cellulose TLC plates (Merck) loaded with 0.5 µg of monophosphate ribonucleotide (rAMP, rCMP, rGMP, UMP) and 300–800 c.p.m. of the ^32^P labeled cap standards (Figure S6 in File S1). The first dimension was developed with solvent A (isobutyric acid: ammonia: water) (66∶1∶33) to separate on negative charge on the phosphate groups and in the second dimension with solvent B (n-propanol: ammonium sulfate: phosphate buffer) (100∶60∶2 V:W:V) to separate on the polarity of nucleotide bases [26], [31], [32].

We performed the following controls to ensure for the specificity of our chromatographies. First, we confirmed that RNAs prepared by omitting the TAP and AP were not eluted from the loading spot (Figure S7A in File S1), indicating that the sRNA preparations did not contain small nucleotides prior digestion. Second, RNA treated with RNAse I followed by T4 PNK treatment migrated away from the loading spot without forming spots corresponding to cap structure (Figure S7B in File S1). Third, no signal corresponding to cap structures was observed when omitting the TAP only (Figure S7C in File S1), confirming that liberation of the caps by TAP treatment was strictly necessary to observe the spot pattern.

We also controlled the activity of the T4 PNK on 5′-hydroxyl nucleosides, since this is the expected product of our enzymatic treatments of the 5′ caps. We used as substrates a mixture of the four monophosphate ribonucleotide (rAMP, rCMP, rGMP, UMP) for labeling reaction without or with prior dephosphorylation (Figure S8 in File S1, panels AB and CD respectively) in the presence of [γ-^32^P]ATP or ATP:[γ-^32^P]ATP, 1∶1 (Figure S8 in File S1, panels AC and BD respectively).

We assigned the spots according to Keith 1999 [33] for position and [34] for chemical structure reference, and quantified the spots with the ImageJ software for fractionated sRNA TLC (Figure S6 in File S1) and Multi-Gauge for immunoprecipitated standards TLC. The intensity of the TLC spots was averaged across replicates after subtracting background intensity and normalizing to the total intensity of all the quantified spots at the same TLC plate. The intensity of spotting origins were saturated in all measurements therefore we were not able to normalize to origin intensity.

### RNA fractionation by HPLC for mass spectroscopy analysis

sRNA was isolated from THP-1 cells using the mirVana kit (Ambion) according to manufacturer’s instructions, and fractionated using micro-reverse phase liquid chromatography with modified condition from (DICKMAN1 and HORNBY2 2006). We used a polystyrene-divinylbenzene PLRP-300 column (3 µm×2 mmID×100 mmL) at 60°C with a flow-rate of 50 µL/min. sRNA was injected at 50 µg/100 µl at time. The RNA fractions were collected at equivalent to 20–50 nt, 50–100 nt, 100–200 nt, and 200–300 nt according to the elution profile of a sRNA ladder (Figure S9 in File S1). We made a gradient of eluent A (100 mM triethylammonium acetate in water TEA-AA) and eluent B (acetonitrile in buffer A, A/CH_3_CN = 60/40). The gradient lasted for 80 min and started with 20% B in A. Concentration of eluent B was extended to 31% in 1 min, followed by an extension to 41% eluent B over 19 min, followed by extension to 70% in 1 min, and elution with 70% eluent B over 9 min. The column was then washed for 40 min with 20% of eluent B. The fractionated RNA was later precipitated using isopropanol and glycogen as a carrier.

### Alignment of Tandem mass spectroscopy

We calibrated and tuned the tandem MS/MS system with the following cap analogs: non-methylated cap analog GpppG (Epicentre), monomethylated cap analog 7mGpppG (Epicentre), dimethylated cap analog 3′-Om,7mGmpppG (3′-0-Me m7G(5′)ppp(5′)G, NEB), and trimethylated cap analog 2,2,7mGpppG (Epicentre). The cap analogs were separated by HPLC and introduced to the spectrometer by electrospray ionization (ESI).

### Enzymatic digestion for mass spectroscopy

RNA fractionated either with PAGE or 150 µg using micro liquid chromatography was digested for three hours at 37°C using RNase T2 (MOB-Funakoshi) and Nuclease P1 from *Penicillium citrinum* (Wako) in 20 mM ammonium acetate buffer pH 5.3. The units used for each enzyme were adjusted according to the manufacturer’s recommendation in order to release the cap core as a 5′-5′ dinucleotide triphosphate despite the possible methylation on the penultimate nucleotide. A C30 monolithic trap column was used to retain and concentrate hydrophobic cap structures from digested RNA samples.

## Results

### Detection of capped sRNA with CAP Trapper

**Figure 1:**
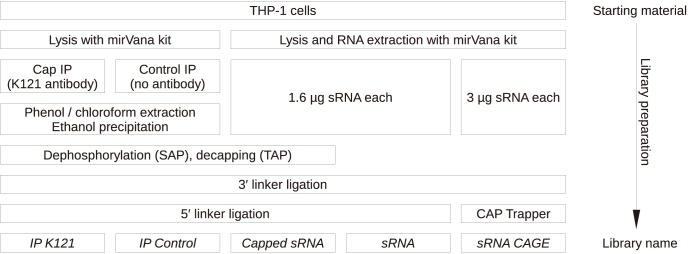
Construction of sequence libraries enriched for capped sRNAs. Step-by-step graphical summary of the construction of the sRNA libraries. The bottom line indicates the library name; in the manuscript, these names are emphasized in italics. The key methods are immunoprecipitation for the IP K121 and IP control libraries, TAP (or mock) treatment for the Capped sRNA and sRNA libraries, and CAP Trapper for the sRNA CAGE libraries.

To detect the presence of caps at the 5′ end of sRNAs, we prepared sequence libraries using three different but complementary procedures (Figure 1). All libraries were prepared in triplicate using sRNA extracted from THP-1 cells. Firstly, we prepared sRNA libraries following a novel protocol combining the CAP trapper chemistry and tag cleavage [14], [29] with sRNA library procedures. In this approach, the reverse-transcription used as template a linker ligated to the 3′ end of sRNAs. We refer to these libraries as sRNA CAGE. These libraries contained in average 35% of reads aligning to loci encoding snRNAs, supporting our intention that this protocol efficiently captured capped RNAs. The other 65% of the reads correspond to multiple annotation categories, the most abundant of which were sections of annotated exons (15%).

### Detection of sRNAs that contain pyrophosphate bonds

A hallmark characteristic of cap structures is the 5′-5′ triphosphate bond between the methylated guanosine and the next nucleotide. In a second set of libraries, we detected RNAs with cap structures by the specific cleavage of these 5′-5′ bonds using tobacco acid pyrophosphatase (TAP) [35]. This cleavage generates a 5′ phosphate that makes the now uncapped sRNAs competent for ligation and inclusion in the sequencing libraries. This done by use of shrimp alkaline phosphatase (SAP) as a step prior to the use of TAP. All naturally occurring 5′-monophosphorylated molecules (e.g. miRNAs) that are in the total RNA pool are rendered incapable for inclusion in capped RNA libraries. We refer to these libraries derived from 5′ capped RNAs as capped sRNA libraries. A separate sequencing library is created for RNAs already possessing a 5′ phosphate in the starting RNA pool, which we called sRNA library.

**Figure 2:**
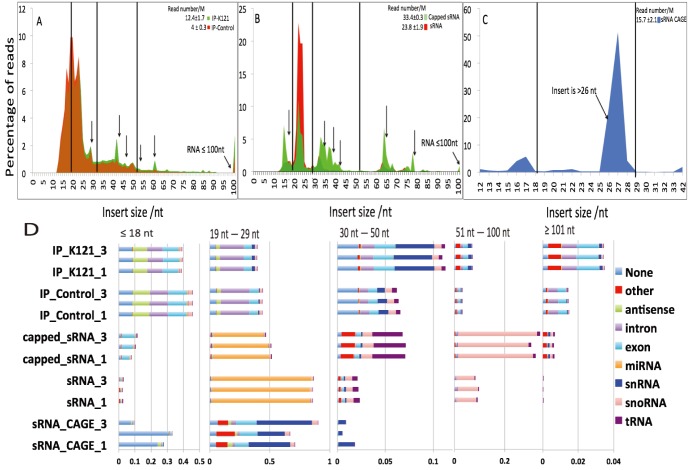
Comparison of libraries identifies enriched sRNA lengths and annotations. A–C: Size profile of the immunoprecipitated (A), TAP-treated (B) and CAP trapped sRNA libraries and their controls. D: Annotations per length range, represented by stacked bar plots for each library replicate. *None* means that no annotation was found, and *other* combines all annotations categories that were not listed individually.

To determine the length of the capped and uncapped sRNAs, we used sequencing read lengths of single end 101 nt. The sRNA libraries showed the expected prominence of miRNAs between 20–24 nt, while the capped sRNA libraries exhibited a more complex pattern, with 54 % of these sRNA (Figure 2) showing lengths greater than 20–24 nts. We then examined in more detail the contents of the libraries by aligning each mapped read to the annotated human genome. We first divided the annotated sets of mapped reads into five size ranges that corresponded to average lengths of different biotypes of annotated sRNAs: 0–18 nts (tiny [ti] RNAs), 19–29 nt (miRNAs), 30–50 nt (TASRs and PASRs), 51–100 nt (tRNAs) and 100–200 nt (snRNA [36], C/D and H/ACA snoRNAs [3]). The most abundant (46%) size range of sRNA biotypes of the capped sRNAs was the 19–29 nt. We also counted 7.4% of the RNAs in the 0–18 nt size range, mostly aligned to exons, which are consistent with them being the tiRNA biotype [12]. 6–8% of the lengths had a size in the 30–50 nt. In that length class, we observed an enrichment for sRNA mapping to tRNA. SnoRNA annotations were prominent in the 50–100 nt range. The relative enrichment of these length classes and biotypes were not observed in the control libraries (sRNA libraries). Altogether, these enrichments show the presence of a diverse collection of sRNAs containing phosphate-phosphate links, a component of RNA cap structures.

### Immunoprecipitation of methylguanosine-capped RNAs from cell extracts

**Figure 3:**
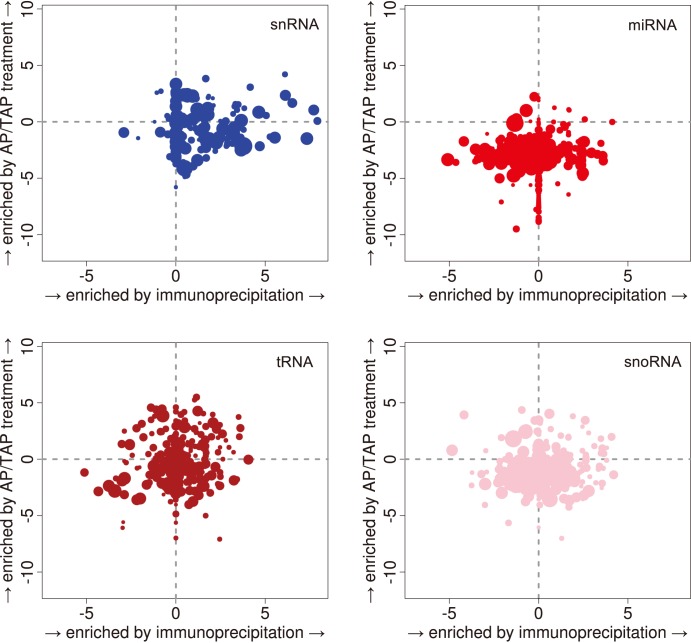
Differential expression analysis of annotated sRNAs. Enrichment of annotated tag clusters in the *IP K121* and *Capped sRNA* libraries respectively to their controls. Horizontal and vertical axis: fold changes in logarithmic scale. Each dot represents one cluster, with a size proportional to its average expression.

Capped RNAs were highly enriched in our sequencing libraries due to the detection of a diol group (sRNA CAGE) or a phosphate-phosphate bond (capped sRNA). As an orthogonal means of detection, we prepared a third set of libraries from RNAs enriched for methylguanosine-capped RNAs by immunoprecipitation with the K121 monoclonal antibody compared to no-antibody controls. We refer to these libraries as IP K121 and IP control respectively. We determined that the K121 antibody had its highest affinity for trimethyl G (2,2,7 mG) caps compared to dimethyl and monomethyl G (2,7 mG and 7 mG, respectively) cap structures (Figure S5 in File S1). The sRNAs present in each of the two libraries were dramatically different in size distribution as well as the annotations they mapped to. This was particularly seen for sRNAs whose lengths were between 12–30 nt (Figure 2A). As expected, in the IP libraries, miRNAs were rarely seen, as was previously reported [12]. Interestingly, fragments of annotated snRNAs in the 30–50 nt range were enriched in the *IP K121* libraries. This enrichment was stronger than in the capped sRNA libraries (Figure 3). The second most enriched sRNAs observed in the *IP K121* libraries were short RNAs (0–18 nt) that mapped within 100 nt of TSSs of annotated gene regions (Figure S10 in File S1). Such short capped RNAs in the IP K121 library are similar to those seen in the capped sRNA libraries and are consistent in the length described for tiRNAs.

### Thin layer chromatography chemically identifies 5′ cap modifications enriched in specific sRNA fractions

**Figure 4:**
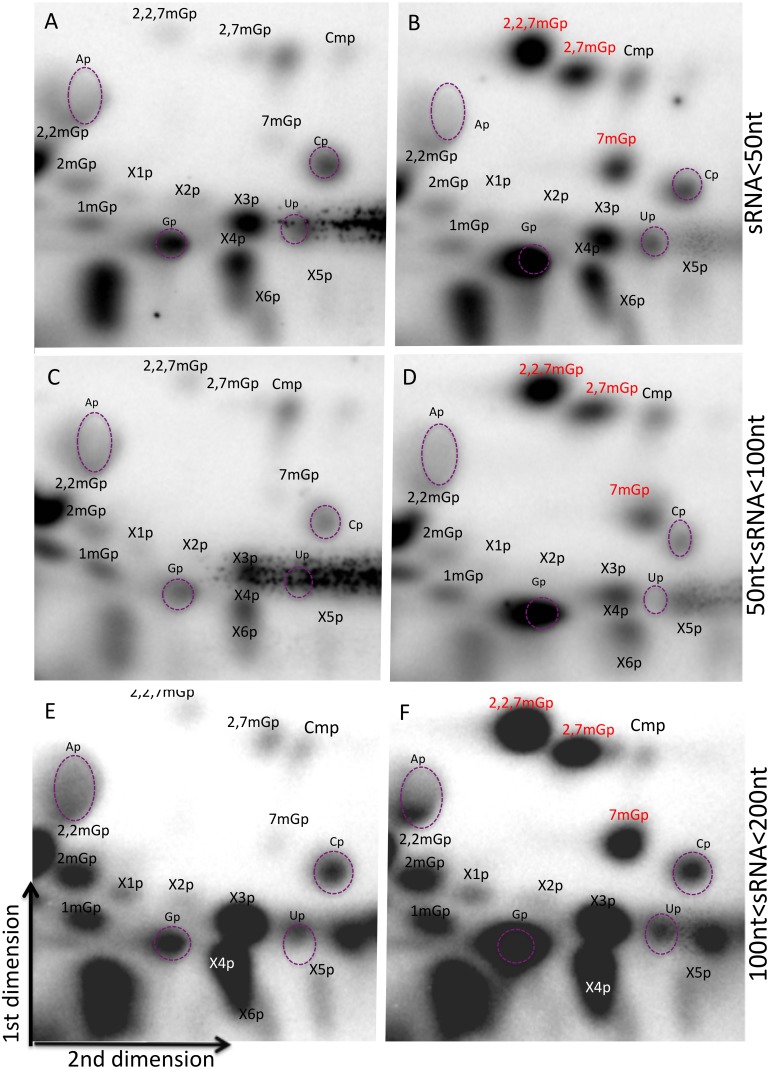
Thin layer chromatography reveals cap structures on sRNAs at all length ranges. A–F: 2D-TLC of radiolabelled cap structures. Long cleavage products corresponding to sRNAs remained immobile on the spotting origin (bottom left corners), and dinucleotides formed large spots at the lower right corner and mid left side, while the cap structures and mononucleotide degradation products migrated further up and right. The first dimension was run using solvent A (see materials and methods) and is displayed from bottom to the top. The second dimension was run using solvent B (left to right). Three size fractions were investigated: <50 nt (A–B), 50–100 nt (C–D), and 100–200 nt (E–F). The position of cold mononucleotides Ap, Gp, Cp, Up detected by UV shadowing is indicated by purple dashed ellipses. B, D, F: chromatographies in presence of radiolabelled standards for Gp, 7 mGp, 2,7 mGp and 2,2,7 mGp. Spots were assigned according to the reference Kieth 1995 or referred to as X.

To further explore the chemical structure of sRNA cap structures, we isolated sRNAs by electrophoresis into three length fractions (1–50 nt, 50–100 nt and 100–200 nt) in triplicate experiments. The cap structures were isolated by incubating each of the size-fractionated sRNAs with TAP. The liberated 5′ cap structures were first dephosphorylated followed by kination with γ-^32^P ATP. The cleavage products were then analyzed using two-dimensional TLC on cellulose plates, eluting the first dimension with isobutyric acid: ammonia: water (66∶1∶33) to separate on nucleic acid bases, and the second dimension with n-propanol: ammonium sulfate: phosphate buffer (100/60/2) to separate on the polarity of molecule. We assigned the spots as published in Kieth *et al.* and Perry *et al.* [33], [37] using radiolabeled cap standards for Gp, 7 mGp, 2,7 mGp, and 2,2,7 mGp, and cold nucleotide monophosphates as a guide (Figure 4). We detected methylguanosine caps in each size sRNA fractions. The spot corresponding to 2,7 mGp could not be unambiguously identified in all samples, as its position varied among replicates and was often split or inhomogeneous in control experiments (Figure S6 in File S1). However, in such cases, we could at least make a preliminary decision based identifying specific spots based on one or two of the replicas.

**Figure 5:**
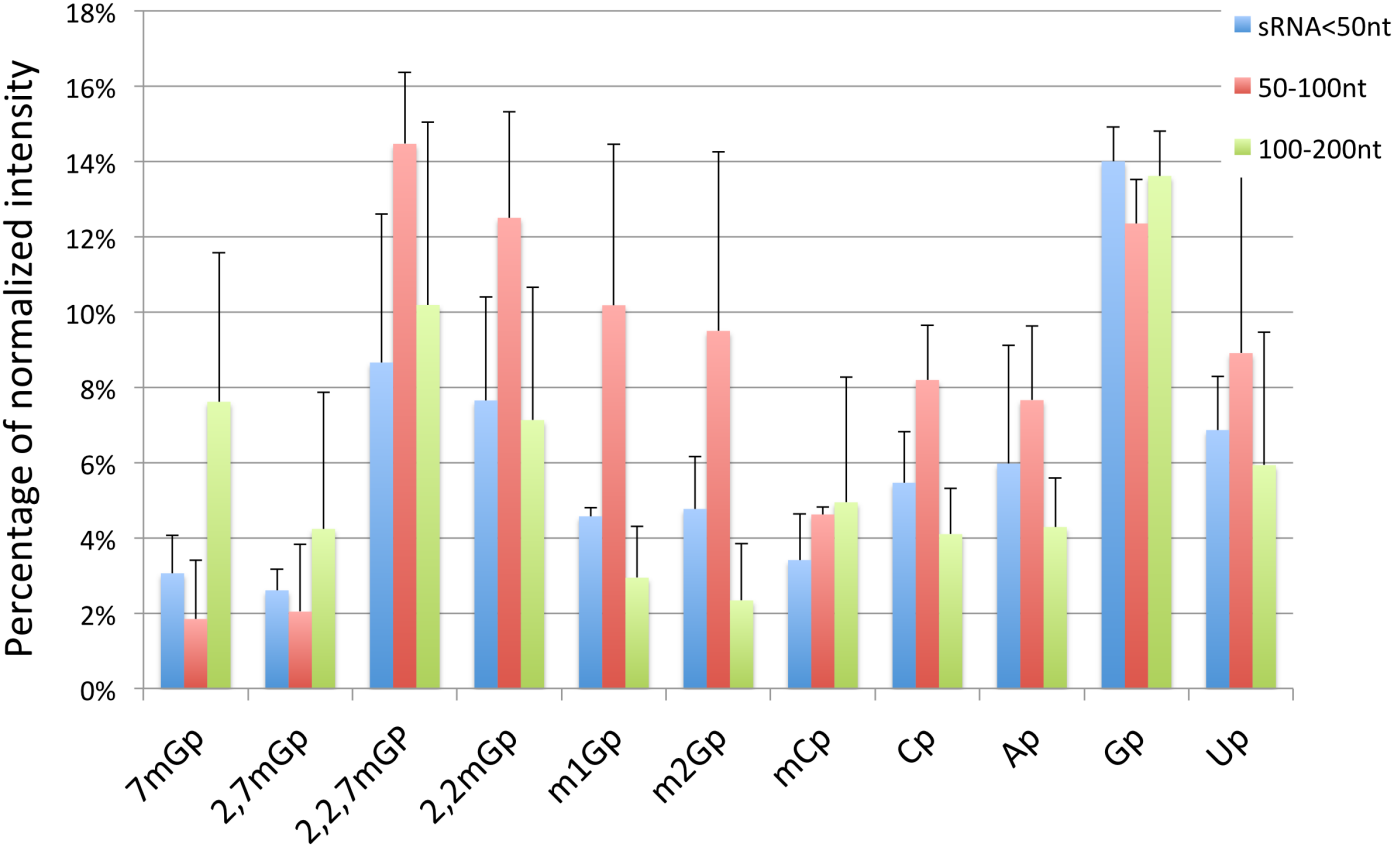
2,2,7 mGp is the most strongly detected methylguanosine in all length ranges. Quantitative analysis of the 2D-TLC, normalized as a percentage of total intensity for each RNA fraction. Error bars represent the standard deviation on three replicates.

In addition to these caps for which we have radiolabelled standards, we also detected spots that correspond to the presence of unlabeled Ap, Up, Cp, Cmp, 1 mGp, 2 mGp, and 2,2 mGp standards. The different cap structures candidates were quantified using intensity percentage after normalization by subtraction of background (Figure 5). In the fraction shorter than 50 nt, the most intense spots corresponded to Gp, 2,2,7 mGp, and 2,2 mGp. Interestingly, in the 50–100 fraction, the most intense spot was 2,2 mGp, a modified nucleotide that is commonly found in tRNAs, for which this fraction was enriched. In addition, despite the fact that those annotated sRNAs biotypes possessing 2,2,7 mG-capped are larger than 100 nt, we also found relatively high amounts of this modified nucleotide in the <50 nt fraction. Overall, the most represented modified nucleotides, in descending order, are Gp (which is the precursor of the canonical cap structures 7 mGp and 2,2,7 mGp), 2,2 mGp, 2,2,7 mGp, 2,7 mGp, and 7 mGp (Figure S11 in File S1 for chemical structures).

### Mass spectroscopic analysis of 5′ end cap structure

Tandem mass spectrometry (MS/MS) was used to provide structure confirmation for the caps that we detected in each of the three length fractions. Each of the fractionated RNA (Figure S9 in File S1) were first digested with RNAse T2 and nuclease P1 to release 5′-5′ di-nucleotide structures, so that in conjunction with the TLC data, we could obtain additional information about the nature and methylation of the first transcribed nucleotide. The sensitivity and specificity of our approach were assessed by characterizing the cap analogs GpppG, 7mGpppG, 3′O,7mGpppG (same molecular weight as 2,7mGpppG), and 2,2,7mGpppG, used to calibrate and tune the tandem MS/MS. We then analyzed the 5′-5′ dinucleotide digested from THP-1 sRNA samples fractionated with PAGE electrophoresis or liquid chromatography.

The tandem mass spectroscopy confirmed several cap structures seen in the TLC experiments (Table 1). In particular, 2,2,7 mG was detected in all sRNA fractions, associated with Am (all fractions), or m6Am, Cm, Gm and Um (in sRNA >100 nt fraction). Modified 2,7 mG and 2,2 mG cap structures were detected along with Am in sRNAs longer than 100 nt. 7 mG was detected with associated with mAm, Gm and Cm in sRNAs shorter than 50 nt and sRNAs longer than 100 nt. Non-methylated guanosine caps were detected in sRNAs shorter than 50 nt and sRNAs longer than 100 nt associated with mAm, A, and G. We detected five dinucleotides cap structures specifically in the sRNA fraction shorter than 50 nt: 7 mG associated with Um, A, or G, and non-methylated guanosine associated with Cm or G. In the fraction longer than 100 nt, potentially new cap structures with unknown modifications were also detected (Table 1).

**Table 1:** Summary of cap structures detected in liquid chromatography-fractionated sRNA.

	sRNA size ranges (nt)					
Structure	20–50	50–100	>100	Calculated mass (M-)	Observed mass (M-)	Error (ppm)
3mGpppAm/Amppp3mG	detected	detected	detected	827.1294	827.1308	1.69
3mGpppmAm/mAmppp3mG	ND	ND	detected	841.1450	841.1466	1.90
2mGpppAm(1)/Amppp2mG(1)	ND	ND	detected	813.1138	813.1157	2.34
2mGpppAm(2)/Amppp2mG(2)	ND	ND	detected	813.1138	813.1157	2.34
mGpppmAm/mAmpppmG	detected	ND	detected	813.1138	813.1157	2.34
3mGpppA/Appp3mG	ND	ND	detected	813.1138	813.1157	2.34
3mGpppCm/Cmppp3mG	ND	ND	detected	803.1184	803.1198	1.74
3mGpppGm/Gmppp3mG	ND	ND	detected	843.1244	843.1261	2.02
3mGpppUm/Umppp3mG	ND	ND	detected	804.1024	804.1041	2.11
mGpppGm/GmpppmG	detected	ND	detected	815.0932	815.0951	2.33
mGpppAm/AmpppmG	ND	ND	detected	799.0982	799.1005	2.88
mGpppCm/CmpppmG	detected	ND	detected	775.0872	775.0885	1.68
mGpppUm/UmpppmG	detected	ND	ND	776.0712	776.0725	1.68
mGpppG/GpppmG	detected	ND	ND	801.078	801.079	1.25
mGpppA/ApppmG	detected	ND	ND	785.083	785.0836	0.76
GpppCm/CmpppG	detected	ND	ND	761.0716	761.073	1.84
GpppGm/GmpppG	detected	ND	ND	801.078	801.0791	1.37
GpppG	detected	ND	detected	787.062	787.0632	1.52
GpppA/ApppG	detected	ND	detected	771.067	771.069	2.59
GpppmAm/mAmpppG	detected	ND	detected	799.0982	799.1006	3.00
Y1pppAm/AmpppY1	ND	ND	detected	-	841.1104	-
2mGpppY2/Y2ppp2mG	ND	ND	detected	-	825.1156	-
Y3pppAm/AmpppY3	ND	ND	detected	-	846.1272	-
Y4pppAm/AmpppY4	ND	ND	detected	-	774.1057	-
Y5pppAm/AmpppY5	ND	ND	detected	-	748.127	-
Y6pppAm/AmpppY6	ND	ND	detected	-	875.154	-
Y7pppAm/AmpppY7	ND	ND	detected	-	875.154	-
Y8pppAm/AmpppY8	ND	ND	detected	-	845.143	-
Y9pppAm/AmpppY9	ND	ND	detected	-	843.1267	-
Y10pppAm/AmpppY10	ND	ND	detected	-	843.1253	-
Y11pppAm/AmpppY11	ND	ND	detected	-	760.0894	-

Abbreviations: ND, not detected; Nm, ribose methylated ribonucleic acid; mN, base monomethylated ribonucleic acid; 2 mN, base dimethylated ribonucleic acid; 3 mN, base trimethylated ribonucleic acid; Yn, unknown structure 2mGpppAm(1) and 2mGpppAm(2) are isomer with different retention time on LC-MS, which may be 2,2mGpppAm and 2,7mGpppAm. Note that the order of the 5′ end nucleotide is not predictable by mass spectroscopy.

Cap structures that were cross-validated by the two techniques are summarized in Table 2. In summary, we detect 2,2,7 mG in all sRNA fractions. We also observed potential cap structures that have not been previously reported, in particular non-methylated guanosine cap and purine methylated guanosine caps. Like the TLC, the MS/MS analysis also uncovered cap structures with unknown ribonucleic acid modifications.

**Table 2:** Summary of cap structures that were cross-validated by 2D-TLC and mass spectroscopy.

5′end structure	Name	Known RNAs with thismodification in human	Biological function ofthis RNA	sRNA fraction with thismodification (nt)
2,2,7mGp	N2,N2,7-trimethylguanosine monophosphate	snoRNA	rRNA modification	20 nt–200 nt
2,2mGp	N2,N2 dimethylguanosine monophosphate	tRNA, rRNA		20 nt–200 nt
2,7mGp	N2,7-dimethylguanosine monphosphate	ND		20 nt–50 nt
7mGp	7-methylguanosine monophosphate	tRNA, rRNA, mRNA		20 nt–50 nt 100 nt
2mGp	2-methylguanosine monophosphate	tRNA		20 nt–200 nt
1mGp	1-methylguanosine monophosphate	tRNA, snRNA	translation	20 nt–50 nt 100 nt
Cmp	2′-O-methylcytidine monophosphate	tRNA, rRNA, snRNA, mRNA	translation, transcription	20 nt–200 nt
Gp	5′-guanosine monophosphate	All		20 nt–200 nt
Cp	5′-cytosine monophosphate	All		20 nt–200 nt
Ap	5′-adenosine monophosphate	All		20 nt–200 nt
Up	5′-uridine monophosphate	All		20 nt–200 nt

*: Unique to this fraction.

**: First time to be reported in mammals.

## Discussion

There is increasingly evidence that loci for both coding and ncRNAs produce various RNA primary transcript and short processed versions of these. Interestingly, cap structures are detected for both long and short RNAs. The conventional functional roles of these cap structures are to assist the capped RNAs in slowing the turn-over, controlling translation [2], [20], [21], and assisting in sub-cellular localization [38], [39]. The cap structures found on sRNA appear to be either added onto the original RNA during transcription (e.g. snoRNAs), or as previously reported, have been added after processing using a novel mechanism [14].

snRNAs were enriched the *IP K121* libraries, suggesting that our immunoprecipitation was at least partially effective. It also enriched the libraries for short tags that aligned to TSS, suggesting that these sRNAs are capped [14]. This result was confirmed by the sRNA CAGE libraries where 26.6% of the tags aligned to TSS (Figure S10 in File S1), similarly to the promoter-associated sRNAs (PASRs) reported by Kapranov et al [15]. This observation support the idea that those transcripts have either 7 mG or 2,2,7 mG caps.

The capped sRNA libraries contained snoRNAs and tRNAs sequences that were remarkably enriched as shorter than 50 nt in length, indicating possible processing of these already sRNAs given that the native transcripts are longer than 70–150 nt. We found that 4% and 32% of the reads aligned to known tRNA and to snoRNA loci, respectively, in these libraries. Nuclear transport of tRNA fragments has been previously reported in several studies on different organisms consistent of a possible functional role for these processed RNAs [40]–[43]. Alternatively, these tRNA fragments could possess 5′ triphosphate originating from the incompleteness of our enzymatic treatments. This possibility would be consistent with the observed presence of miRNA sequences in the capped sRNA libraries.

By pyrophosphatase cleavage and TLC, we detected a series of 5′ monophosphate ribonucleotides which were reported previously as internal modified bases (1 mG, 2 mG, 2,2 mG, Cm) in tRNAs and rRNAs [44], [45]. This observation raises the the possibility that these modified nucleotides are the result of non-specific degradation of the analyzed sRNA. However, this possibility is unlikely since accidental degradation to single nucleotides is unlikely as alkaline hydrolysis requires incubation for at least 12 h with a strong base [46] and enzymatic digestion requires the combination of different RNases [47], [48], both of which being very extreme conditions comparing to our procedure for cap isolation. Importantly, these simple modified bases were not confirmed in our mass spectroscopy experiments, suggesting that their relative abundance is likely to be lower than the methylguanosine caps and under the sensitivity limits for mass spectroscopy.

To our knowledge, we report here for the first time that 2,7 dimethylguanosine in human sRNAs at reasonably abundant amounts. In humans, 7 mG caps are hypermethylated by the guanine-N2 methyltransferase TGS-1 (Trimethylguanosine Synthase 1), which is inefficient in methylating other substrates, such as non-methylated G or 2 mG [7]. The kinetic of conversation from 7 mG to 2,2,7 mG has not as yet been reported to occur *in vivo*. 7 mG and 2,2,7 mG cap structures were double confirmed by 2D-TLC analysis and tandem mass spectroscopy in sRNA shorter than 50 nt. 2,2,7 mG was detected in all sRNA fractions, associated with Am (all fractions), or m6Am, Cm, Gm and Um (<200 nt fraction). We also found typical cap structure structures in RNAs shorter than 50 nt (7mGpppUm, 7mGpppA, 7mGpppG).

A study on human TGS-1 *in vitro* showed the conversion of 90% of 7mGpp substrate to 2,7mGpp after 5 min of incubation, while the conversion of more than 50% of 2,7mGpp to 2,2,7mGpp required 15 min of treatment [49]. The efficiency of TGS-1 on both substrates was also proven by Benarroch et al [7], who reported similar catalytic efficiencies for the 2,7 mG and 2,2,7 mG synthase reactions. Thus, given their short half-life, it is tempting to speculate that the 2,7 mG caps that we detected are not intermediate products. We observed this modified cap structure using TLC and mass spectrometry on sRNA longer than 100 nt.

2,7 mG cap was reported previously in Sinbis virus 28S rRNA and protozoan parasite like *Giardia lamblia* which has cap guanine-N2 methyltransferase TGS-2 enzymes but not TGS-1 [50]. This is consistent with not only the possibility of the existence of individual transcripts that carry this type of modification but also with the possibility of the presence of uncharacterized TGS-2 enzyme in human cells or pathways that stop after the first methylation by TGS-1.

Caps are classified as *type 0*, *I*, *II*, etc. according to the number 2′-O-methylated nucleotides that follow the 5′-5′ bond. In mRNAs, *type I* (or higher) caps increase the translation efficiency [51], affect the interaction with eIF4E [52], and the transport to the cytoplasm [38], [39]. Mass spectrometry allowed us detect 2′-O methylation on the first nucleotide and therefore distinguish *type 0* from the other types. In this study, surprisingly we found multiple *type 0* cap structures (mGpppC, 7mGpppG, GpppG, GpppA, and 7mGpppA) in RNA length fractions shorter than 50 nt. Thus, these sRNAs cannot be degradation products of mRNA 5′ end, suggesting other mechanisms such as cleavage and recapping from longer precursor RNAs. To the best of our knowledge, this is the first report of *type 0* cap structure in human RNAs.

We also found the 5′-5′ dinucleotides GpppmAm and GpppmC, for which the TLC data suggests that G is the outer 5′ cap nucleotide and Am or mC are the next nucleotides, because in all length fractions Gp was the most abundant nucleotide by TLC. Interestingly, our TLC results also showed a spot that may be mCp, leaving open the possibility of a mCpppG cap. This result would be unusual and in need of additional confirmation.

Our study also suggests the presence of additional uncharacterized cap structures due to the absence of a reference compound in TLC and thus making the structural information obtained from mass spectroscopic data difficult to interpret. While performing positive control TLC analysis to test the ability of T4 PNK to phosphorylate nucleosides (Figure S8 in File S1) we could observe two spots similar to what we call unknowns X1 and X4. Those spots may reflect the possibility of the phosphorylation of both 5′ and 3′ position of the nucleoside. However, formal validation of this possibility awaits further testing.

Finally, combining cDNA cloning of sRNAs, precipitation by anti-methylguanosine antibodies, thin layer chromatography and mass spectroscopy, we observe the unambiguous presence of caps on sRNAs, not only in the size range corresponding to the annotated snRNAs, but also in the size range of tRNAs (∼75 nt) and in the sRNAs shorter than 50 nt. In this size range, where we found sRNAs aligning to the transcription start and termination sites in known genes. We also found *type 0* variants of these caps, which may be produced by cytoplasmic capping. This analysis approach opens the possibility of highly promiscuous 5′ end modifications of both long and short RNAs or a novel landscape of structural cap modifications with possible novel biological roles. Further investigation of the biogenesis of these RNAs will be needed to understand if they are the direct product of short-range transcription or if they are the cleavage product of longer templates.

## Supporting Information

File S1 **Contains the supplementary figures and their legends.**
(PDF)

## References

[1] Shimotohno K, Kodama Y, Hashimoto J, Miura KI (1977) Importance of 5′-terminal blocking structure to stabilize mRNA in eukaryotic protein synthesis. Proc Natl Acad Sci U S A 74: 2734–2738. 197518 10.1073/pnas.74.7.2734 PMC431268

[2] Banerjee AK (1980) 5′-terminal cap structure in eucaryotic messenger ribonucleic acids. Microbiol Mol Biol Rev 44: 175–205. 10.1128/mr.44.2.175-205.1980 PMC373176 6247631

[3] Matera AG, Terns RM, Terns MP (2007) Non-coding RNAs: lessons from the small nuclear and small nucleolar RNAs. Nat Rev Mol Cell Biol 8: 209–220. 17318225 10.1038/nrm2124

[4] Singh R, Reddy R (1989) Gamma-monomethyl phosphate: a cap structure in spliceosomal U6 small nuclear RNA. Proc Natl Acad Sci U S A 86: 8280–8283. 2813391 10.1073/pnas.86.21.8280 PMC298264

[5] O’Mullane L, Eperon IC (1998) The Pre-mRNA 5′ Cap Determines Whether U6 Small Nuclear RNA Succeeds U1 Small Nuclear Ribonucleoprotein Particle at 5′ Splice Sites. Mol Cell Biol 18: 7510–7520. 9819436 10.1128/mcb.18.12.7510 PMC109331

[6] Hsuchen C-C, Dubin DT (1976) Di- and trimethylated congeners of 7-methylguanine in Sindbis virus mRNA. Nature 264: 190–191. 995206 10.1038/264190a0

[7] Benarroch D, Jankowska-Anyszka M, Stepinski J, Darzynkiewicz E, Shuman S (2010) Cap Analog Substrates Reveal Three Clades of Cap Guanine-N2 Methyltransferases with Distinct Methyl Acceptor Specificities. RNA 16: 211–220. 19926722 10.1261/rna.1872110 PMC2802030

[8] Kawaji H, Nakamura M, Takahashi Y, Sandelin A, Katayama S, et al (2008) Hidden layers of human small RNAs. BMC Genomics 9: 157. 18402656 10.1186/1471-2164-9-157 PMC2359750

[9] Carninci P, Kasukawa T, Katayama S, Gough J, Frith MC, et al (2005) The Transcriptional Landscape of the Mammalian Genome. Science 309: 1559–1563. 16141072 10.1126/science.1112014

[10] Imanishi T, Itoh T, Suzuki Y, O’Donovan C, Fukuchi S, et al (2004) Integrative Annotation of 21,037 Human Genes Validated by Full-Length cDNA Clones. PLoS Biol 2: e162. 15103394 10.1371/journal.pbio.0020162 PMC393292

[11] Meyer LR, Zweig AS, Hinrichs AS, Karolchik D, Kuhn RM, et al (2012) The UCSC Genome Browser database: extensions and updates 2013. Nucleic Acids Res 41: D64–D69. 23155063 10.1093/nar/gks1048 PMC3531082

[12] Taft RJ, Glazov EA, Lassmann T, Hayashizaki Y, Carninci P, et al (2009) Small RNAs Derived from snoRNAs. RNA 15: 1233–1240. 19474147 10.1261/rna.1528909 PMC2704076

[13] Lee YS, Shibata Y, Malhotra A, Dutta A (2009) A novel class of small RNAs: tRNA-derived RNA fragments (tRFs). Genes Dev 23: 2639–2649. 19933153 10.1101/gad.1837609 PMC2779758

[14] Fejes-Toth K, Sotirova V, Sachidanandam R, Assaf G, Hannon GJ, et al (2009) Post-transcriptional processing generates a diversity of 5′-modified long and short RNAs. Nature 457: 1028–1032. 19169241 10.1038/nature07759 PMC2719882

[15] Kapranov P, Ozsolak F, Kim SW, Foissac S, Lipson D, et al (2010) New class of gene-termini-associated human RNAs suggests a novel RNA copying mechanism. Nature 466: 642–646. 20671709 10.1038/nature09190 PMC3058539

[16] McCue AD, Slotkin RK (2012) Transposable element small RNAs as regulators of gene expression. Trends Genet. 28(12): 616–623. 10.1016/j.tig.2012.09.001 23040327

[17] Qureshi IA, Mehler MF (2012) Emerging roles of non-coding RNAs in brain evolution, development, plasticity and disease. Nat Rev Neurosci 13: 528–541. 22814587 10.1038/nrn3234 PMC3478095

[18] Mazières J, Catherinne C, Delfour O, Gouin S, Rouquette I, et al (2013) Alternative Processing of the U2 Small Nuclear RNA Produces a 19–22nt Fragment with Relevance for the Detection of Non-Small Cell Lung Cancer in Human Serum. PLoS ONE 8: e60134. 23527303 10.1371/journal.pone.0060134 PMC3603938

[19] Otsuka Y, Kedersha NL, Schoenberg DR (2009) Identification of a cytoplasmic complex that adds a cap onto 5′-monophosphate RNA. Mol Cell Biol 29: 2155–2167. 19223470 10.1128/MCB.01325-08 PMC2663312

[20] Ro-Choi TS (1999) Nuclear snRNA and nuclear function (discovery of 5′ cap structures in RNA). Crit Rev Eukaryot Gene Expr 9: 107–158. 10445153 10.1615/critreveukargeneexpr.v9.i2.20

[21] Ro-Choi TS, Choi YC (2012) Chemical Approaches for Structure and Function of RNA in Postgenomic Era. J Nucleic Acids 2012: 369058. 22347623 10.1155/2012/369058 PMC3278928

[22] Takahashi H, Kato S, Murata M, Carninci P (2012) CAGE (Cap Analysis of Gene Expression): A Protocol for the Detection of Promoter and Transcriptional Networks. In: Deplancke B, Gheldof N, editors. Gene Regulatory Networks. Totowa, NJ: Humana Press, Vol. 786: 181–200. 10.1007/978-1-61779-292-2_11 PMC4094367 21938627

[23] Kawano M, Kawazu C, Lizio M, Kawaji H, Carninci P, et al (2010) Reduction of non-insert sequence reads by dimer eliminator LNA oligonucleotide for small RNA deep sequencing. BioTechniques 49: 751–755. 20964636 10.2144/000113516

[24] Li H, Handsaker B, Wysoker A, Fennell T, Ruan J, et al (2009) The Sequence Alignment/Map format and SAMtools. Bioinformatics 25: 2078–2079. 19505943 10.1093/bioinformatics/btp352 PMC2723002

[25] Quinlan AR, Hall IM (2010) BEDTools: a flexible suite of utilities for comparing genomic features. Bioinformatics 26: 841–842. 20110278 10.1093/bioinformatics/btq033 PMC2832824

[26] Ruan J, Ullu E, Tschudi C (2007) Characterization of the Trypanosoma brucei cap hypermethylase Tgs1. Mol Biochem Parasitol 155: 66–69. 17610965 10.1016/j.molbiopara.2007.05.008 PMC2075351

[27] Girard C, Verheggen C, Neel H, Cammas A, Vagner S, et al (2008) Characterization of a Short Isoform of Human Tgs1 Hypermethylase Associating with Small Nucleolar Ribonucleoprotein Core Proteins and Produced by Limited Proteolytic Processing. J Biol Chem 283: 2060–2069. 18039666 10.1074/jbc.M704209200

[28] Krainer AR (1988) Pre-mRNA splicing by complementation with purified human UI, U2, U4/U6 and U5 snRNPs. Nucleic Acids Res 16: 9415–9429. 3141901 10.1093/nar/16.20.9415 PMC338753

[29] Hartmann RK, Bindereif A, Schon A, Westhof E (2009) Handbook of RNA biochemistry student edition. Weinheim: Wiley-VCH-Verl.pp133–150.

[30] Bochner BR, Ames BN (1982) Complete analysis of cellular nucleotides by two-dimensional thin layer chromatography. J Biol Chem 257: 9759–9769. 6286632

[31] Simoes-Barbosa A, Louly C, Franco OL, Rubio MA, Alfonzo JD, et al (2008) The Divergent Eukaryote Trichomonas Vaginalis Has an m7G Cap Methyltransferase Capable of a Single N2 Methylation. Nucleic Acids Res 36: 6848–6858. 18957443 10.1093/nar/gkn706 PMC2588526

[32] Grosjean H, Droogmans L, Roovers M, Keith G (2007) Detection of Enzymatic Activity of Transfer RNA Modification Enzymes Using Radiolabeled tRNA Substrates. In: Academic Press, Vol. Volume Jonatha M Gott, editor. Methods in Enzymology. 425: 55–101. 10.1016/S0076-6879(07)25003-7 17673079

[33] Keith G (1995) Mobilities of modified ribonucleotides on two-dimensional cellulose thin-layer chromatography. Biochimie 77: 142–144. 7599271 10.1016/0300-9084(96)88118-1

[34] Limbach PA, Crain PF, Pomerantz SC, McCloskey JA (1995) structures of posttranscriptional modified nucleosides from RNA. Biochimie 77: 135–138. 7541251 10.1016/0300-9084(96)88116-8

[35] Huang F, Yarus M (1997) 5′-RNA Self-Capping from Guanosine Diphosphate. Biochemistry (Mosc) 36: 6557–6563. 10.1021/bi970475b 9184134

[36] Lerner MR, Boyle JA, Mount SM, Wolin SL, Steitz JA (1980) Are snRNPs involved in splicing? Nature 283: 220–224. 7350545 10.1038/283220a0

[37] Perry KL, Watkins KP, Agabian N (1987) Trypanosome mRNAs have unusual “cap 4” structures acquired by addition of a spliced leader. Proc Natl Acad Sci U S A 84: 8190–8194. 3120186 10.1073/pnas.84.23.8190 PMC299507

[38] Camper SA, Albers RJ, Coward JK, Rottman FM (1984) Effect of undermethylation on mRNA cytoplasmic appearance and half-life. Mol Cell Biol 4: 538–543. 6201720 10.1128/mcb.4.3.538-543.1984 PMC368733

[39] Dimock K, Stoltzfus CM (1979) Processing and function of undermethylated chicken embryo fibroblast mRNA. J Biol Chem 254: 5591–5594. 221474

[40] Zaitseva L, Myers R, Fassati A (2006) tRNAs Promote Nuclear Import of HIV-1 Intracellular Reverse Transcription Complexes. PLoS Biol 4: e332. 17020411 10.1371/journal.pbio.0040332 PMC1584419

[41] Simos G, Hurt E (1999) Transfer RNA biogenesis: A visa to leave the nucleus. Curr Biol 9: R238–R241. 10209112 10.1016/s0960-9822(99)80152-3

[42] Arts G-J, Fornerod M, Mattaj lain W (1998) Identification of a nuclear export receptor for tRNA. Curr Biol 8: 305–314. 9512417 10.1016/s0960-9822(98)70130-7

[43] Hanada T, Weitzer S, Mair B, Bernreuther C, Wainger BJ, et al (2013) CLP1 links tRNA metabolism to progressive motor-neuron loss. Nature 495: 474–480. 23474986 10.1038/nature11923 PMC3674495

[44] Iwanami Y, Brown GM (1968) Methylated bases of ribosomal ribonucleic acid from HeLa cells. Arch Biochem Biophys 126: 8–15. 5671075 10.1016/0003-9861(68)90553-5

[45] Rozenski J, Crain PF, McCloskey JA (1999) The RNA Modification Database: 1999 update. Nucleic Acids Res 27: 196–197 doi:10.1093/nar/27.1.196 9847178 10.1093/nar/27.1.196 PMC148133

[46] Lipkin D, Talbert PT, Cohn M (1954) The Mechanism of the Alkaline Hydrolysis of Ribonucleic Acids. J Am Chem Soc 76: 2871–2872.

[47] Helm M, Florentz C, Chomyn A, Attardi G (1999) Search for Differences in Post-Transcriptional Modification Patterns of Mitochondrial DNA-Encoded Wild-Type and Mutant Human tRNALys and tRNALeu(UUR). Nucleic Acids Res 27: 756–763. 9889270 10.1093/nar/27.3.756 PMC148244

[48] Helm M (2006) Post-transcriptional nucleotide modification and alternative folding of RNA. Nucleic Acids Res 34: 721–733. 16452298 10.1093/nar/gkj471 PMC1360285

[49] Hausmann S, Zheng S, Costanzo M, Brost RL, Garcin D, et al (2008) Genetic and Biochemical Analysis of Yeast and Human Cap Trimethylguanosine Synthase. Functional Overlap of 2,2,7- Trimethyguanosine Caps, Small Nuclear Ribonucleoprotein Components, Pre-mRNA Splicing Factors, and RNA Decay Pathways. J Biol Chem 283: 31706–31718. 18775984 10.1074/jbc.M806127200 PMC2581544

[50] Hausmann S, Shuman S (2005) Giardia Lamblia RNA Cap Guanine-N2 Methyltransferase (Tgs2). J Biol Chem 280: 32101–32106. 16046409 10.1074/jbc.M506438200

[51] Kuge H, Brownlee GG, Gershon PD, Richter JD (1998) Cap ribose methylation of c-mos mRNA stimulates translation and oocyte maturation in Xenopus laevis. Nucleic Acids Res 26: 3208–3214. 9628920 10.1093/nar/26.13.3208 PMC147664

[52] Zuberek J, Wyslouch-Cieszynska A, Niedzwiecka A, Dadlez M, Stepinski J, et al (2003) Phosphorylation of eIF4E attenuates its interaction with mRNA 5′ cap analogs by electrostatic repulsion: Intein-mediated protein ligation strategy to obtain phosphorylated protein. RNA 9: 52–61. 12554876 10.1261/rna.2133403 PMC1370370

