# Supplementary material for: Multiplicity of 5′ Cap Structures Present on Short RNAs
Source: PLoS One. 2014 Jul 31;9(7):e102895. doi: 10.1371/journal.pone.0102895 (PMC4117478; doi:10.1371/journal.pone.0102895)

Figure S1: The TAP enzymes currently available on the market are not recombinant, and carry an uncharacterized RNase activity of variable intensity depending on the maker (this figure and unpublished observations). We tested RNAse activity of Epicenter TAP, using 1, 2, 3, 4, 5, 10, and 15 U of enzyme and 500 ng of mouse liver total RNA in 50 µl reaction volume and incubated for 60 min at 37 °C followed by phenol/chloroform extraction and ethanol precipitation. The RNA profile was checked on a Agilent BioAnalyzer with a pico RNA kit.

The sRNA fraction kept the same size profile at the highest quantity of TAP (15 U) compared to the untreated control (upper panels). While the RIN value and the 18S/28S ratio decreased when the quantity of TAP increased, the proportion between sRNA and RNA longer than 200 nt remained stable.

In this work, we used 1 U TAP (Epicenter) for 5 µg RNA, and in the hypothesis that the RNase activity is caused by a contamination during the purification of the enzyme, our reactions contained an RNase inhibitor. Our control experiment shows that at 1 U TAP and even higher quantities, there is no sign of extensive degradation that would produce observable quantities of sRNAs.

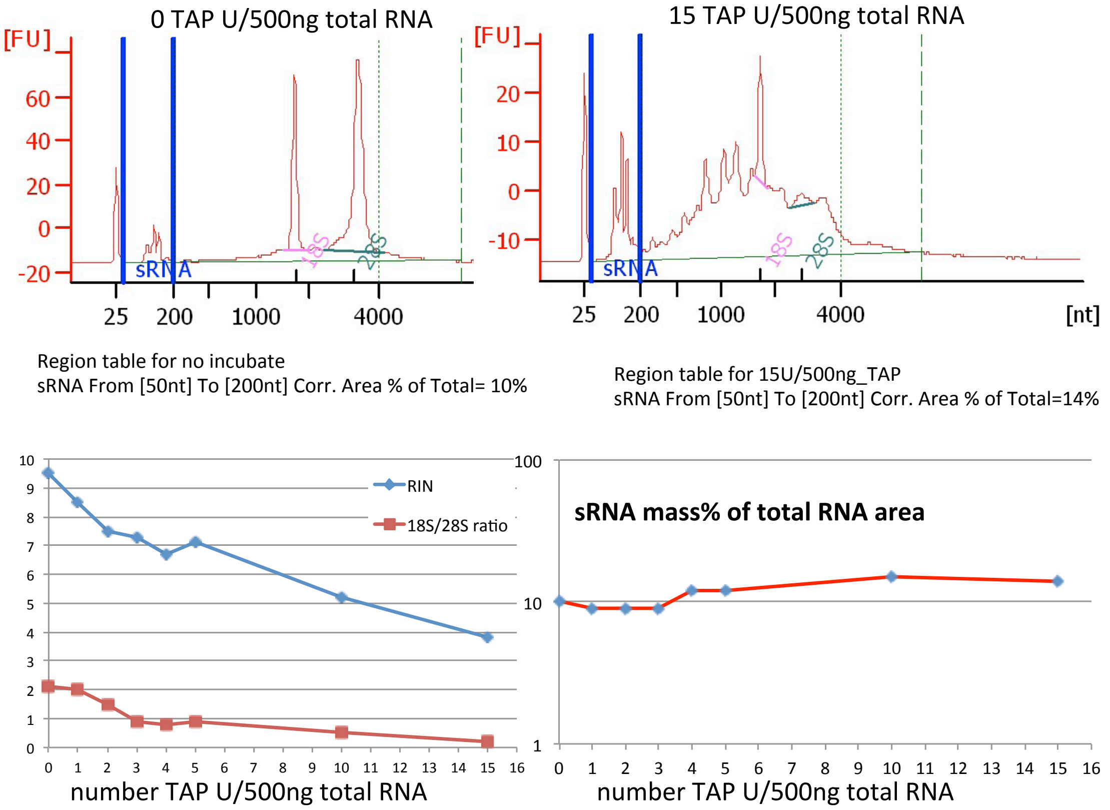

Figure S2: sRNA fractionation on 15% denaturing PAGE. The size fractions were collected by cutting the gel into three slices (< 50 nt, 50–100 nt, 100–200 nt) according to a RNA molecular weight marker (RNA lowII, DynaMarker).

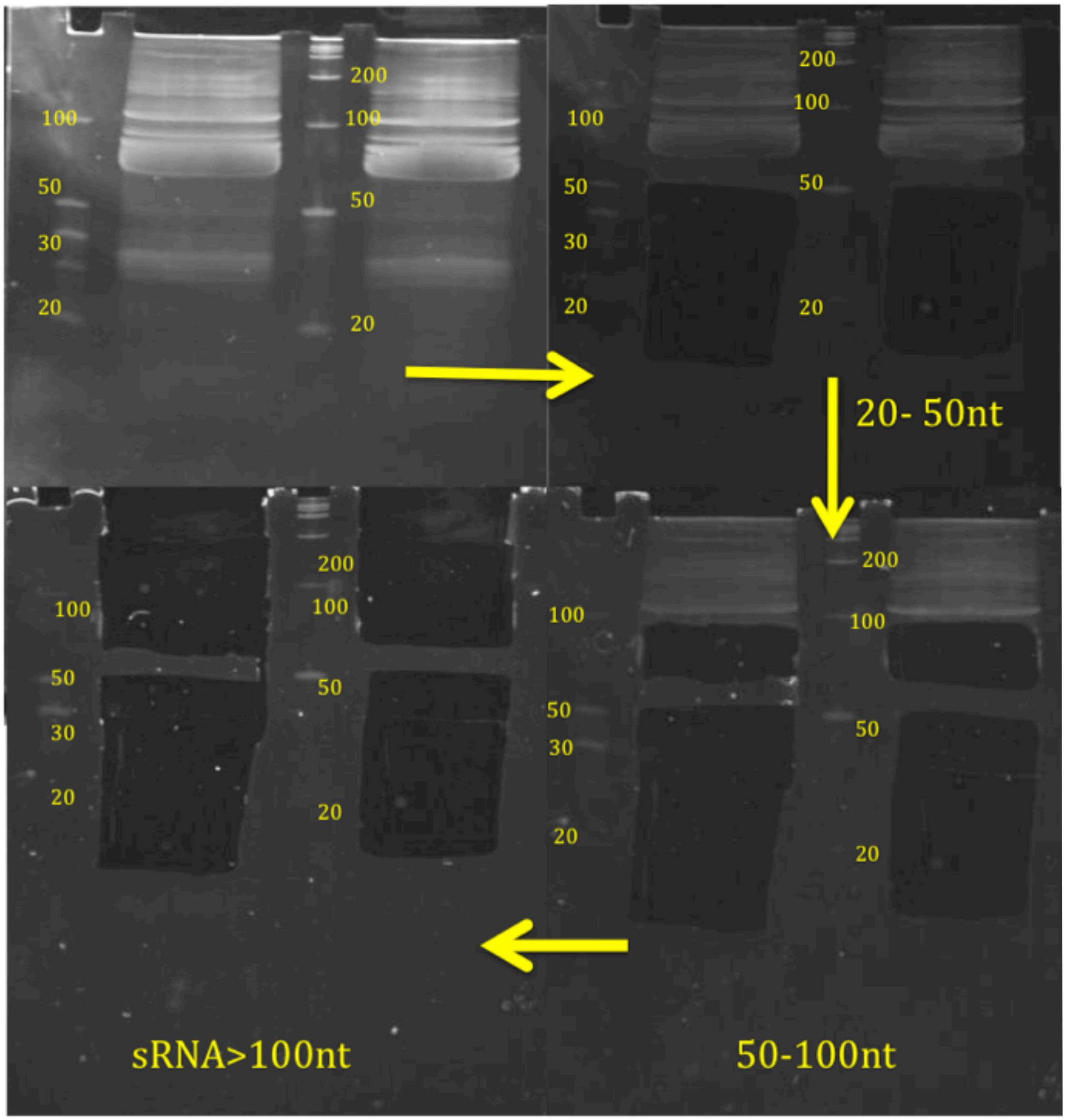

Figure S3: Replicates of the TLC of immunoprecipitated cap standards. See Figure S4, S5 for details

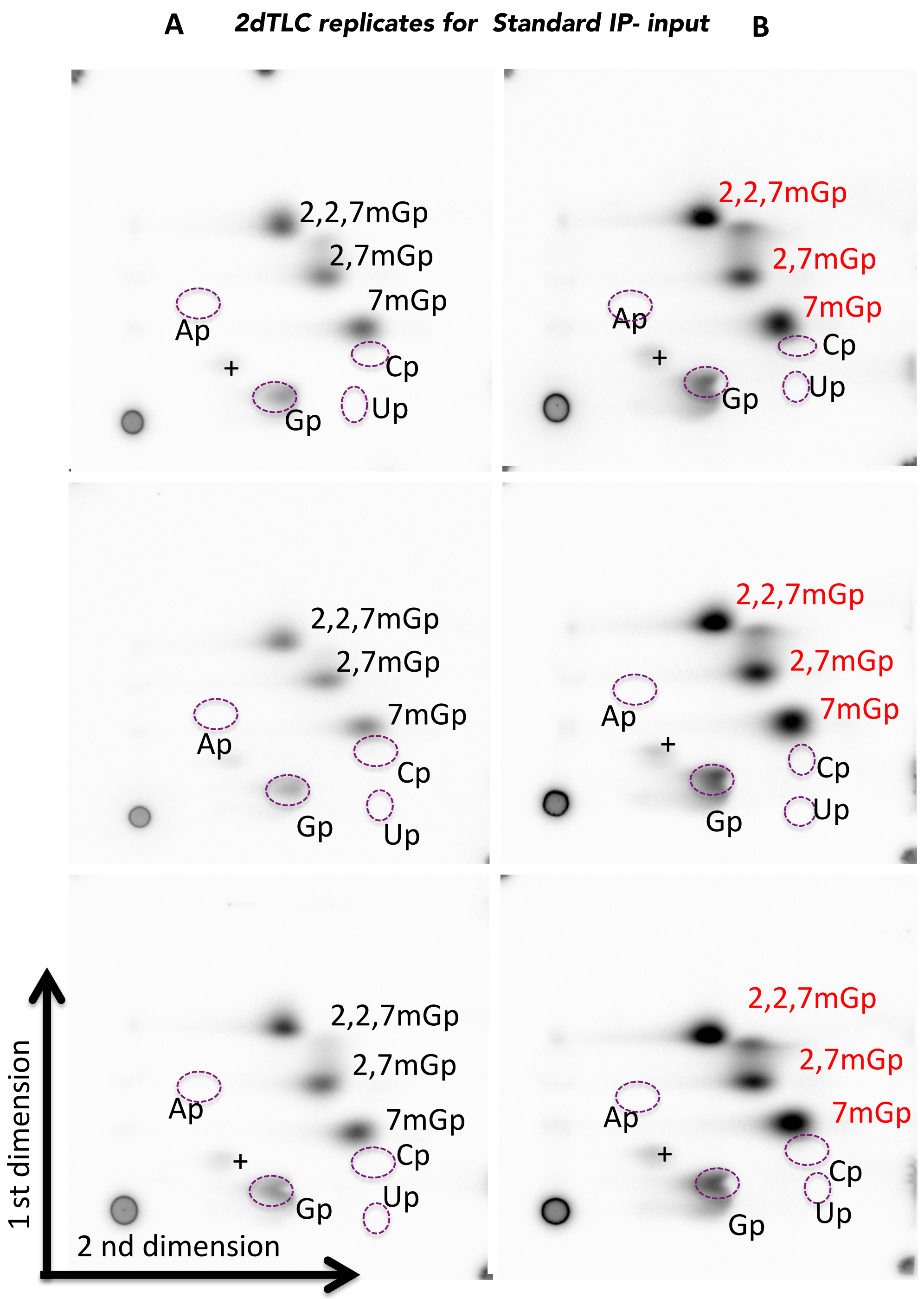

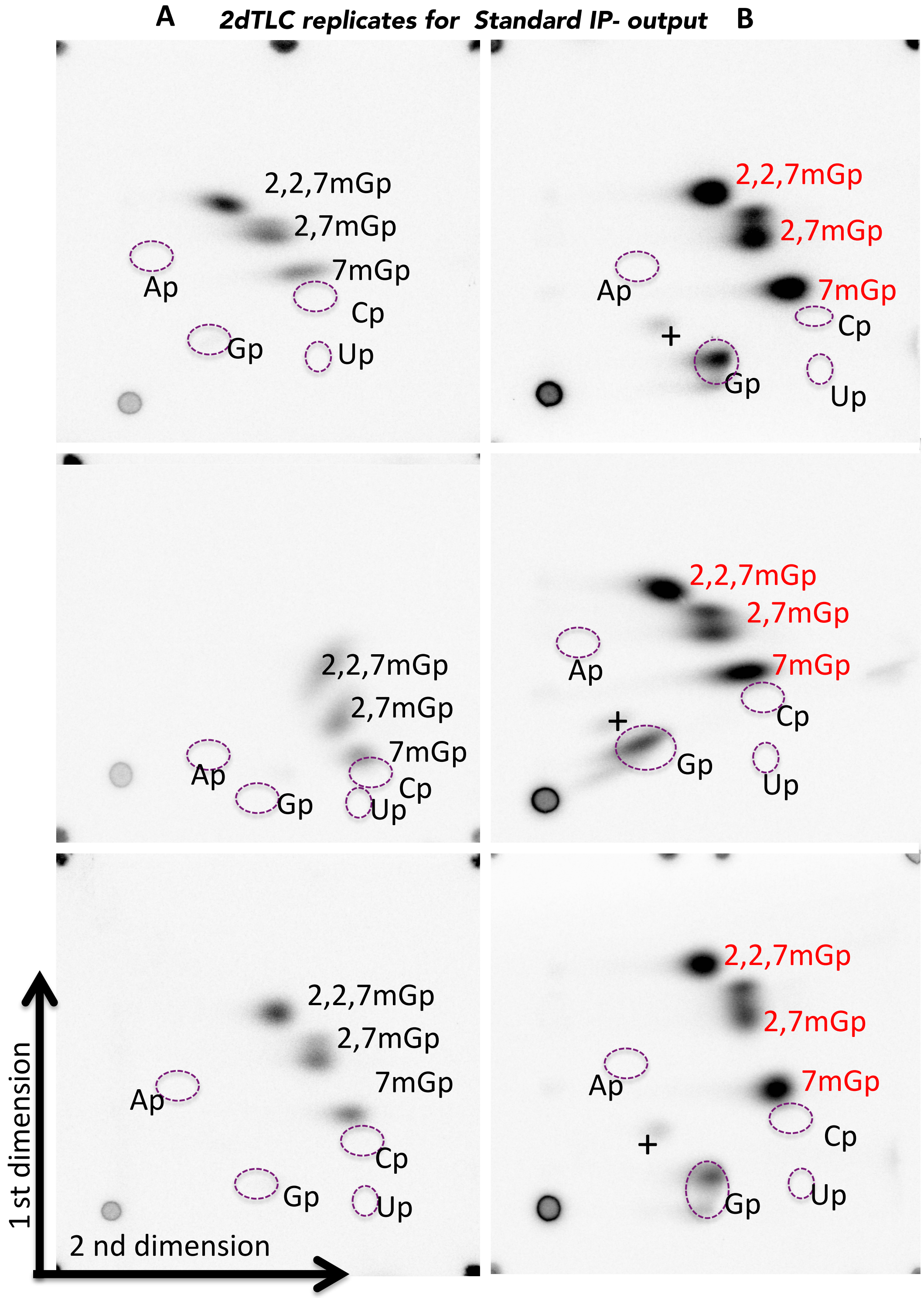
A
2dTLC replicates for Standard IP- output
B
2,2,7mGp
2,7mGp
7mGp
Ap
Cp
Gp
Up
+
1 st dimension
2 nd dimension

Figure S4: Immunoprecipitation of cap nucleotides with the K121 antibody. A: 1D-TLC with solvent A of the IP input, IP output and no-antibody control. *: mark indicate the spotting position of the control. +: artifact. The position of cold mononucleotides Ap, Gp, Cp, Up detected by UV shadowing is indicated by purple dashed ellipses. B: radioactive count of IP control, output and input, (in cpm/µl). C: Recovery of the radioactivity in each IP replicate and in the no-antibody control.

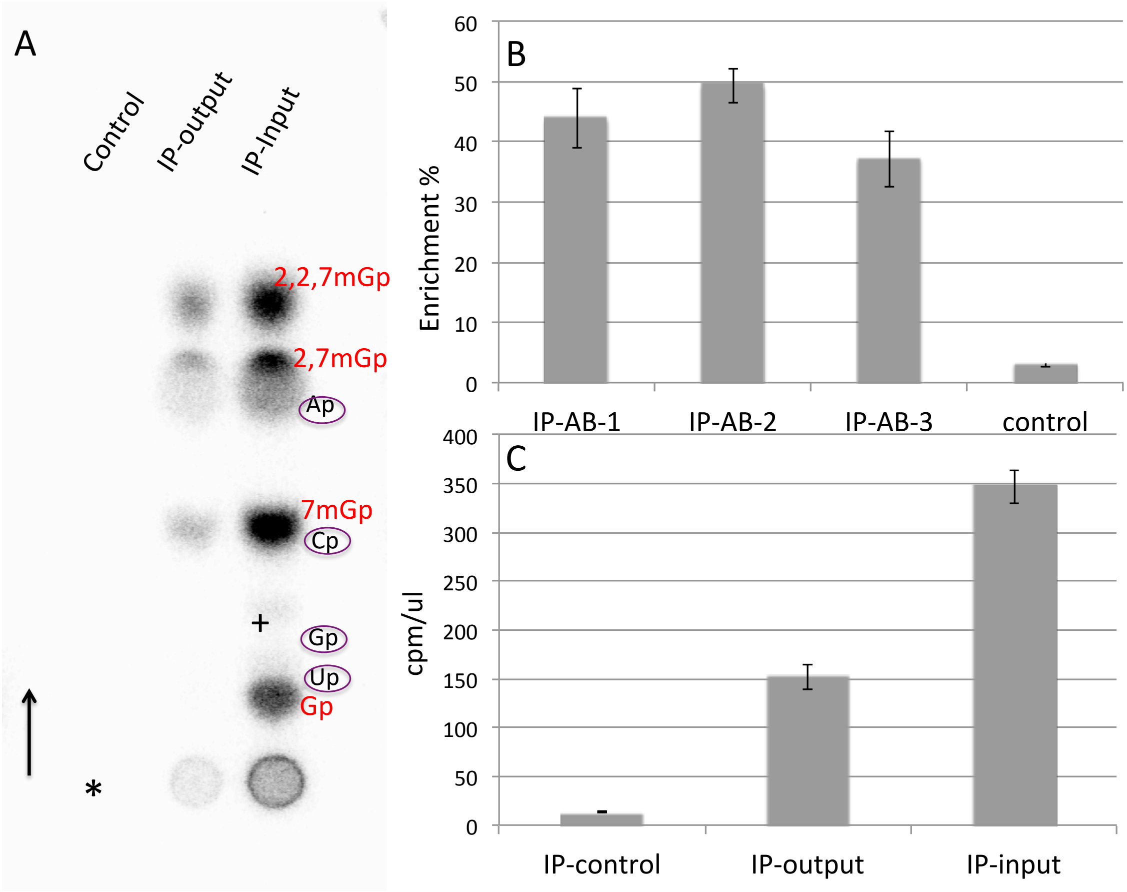

Figure S5: Immunoprecipitation of cap nucleotides with the K121 antibody. A and B: input without (A) and with (B) radiolabelled standards Gp, 7mGp, 2,7mGp and 2,2,7mGp. C and D: immunoprecipitation without (C) and with (D) radiolabelled standards. E and F: quantification of the 2D-TLC for each cap standard in the IP input (E) or output (F)

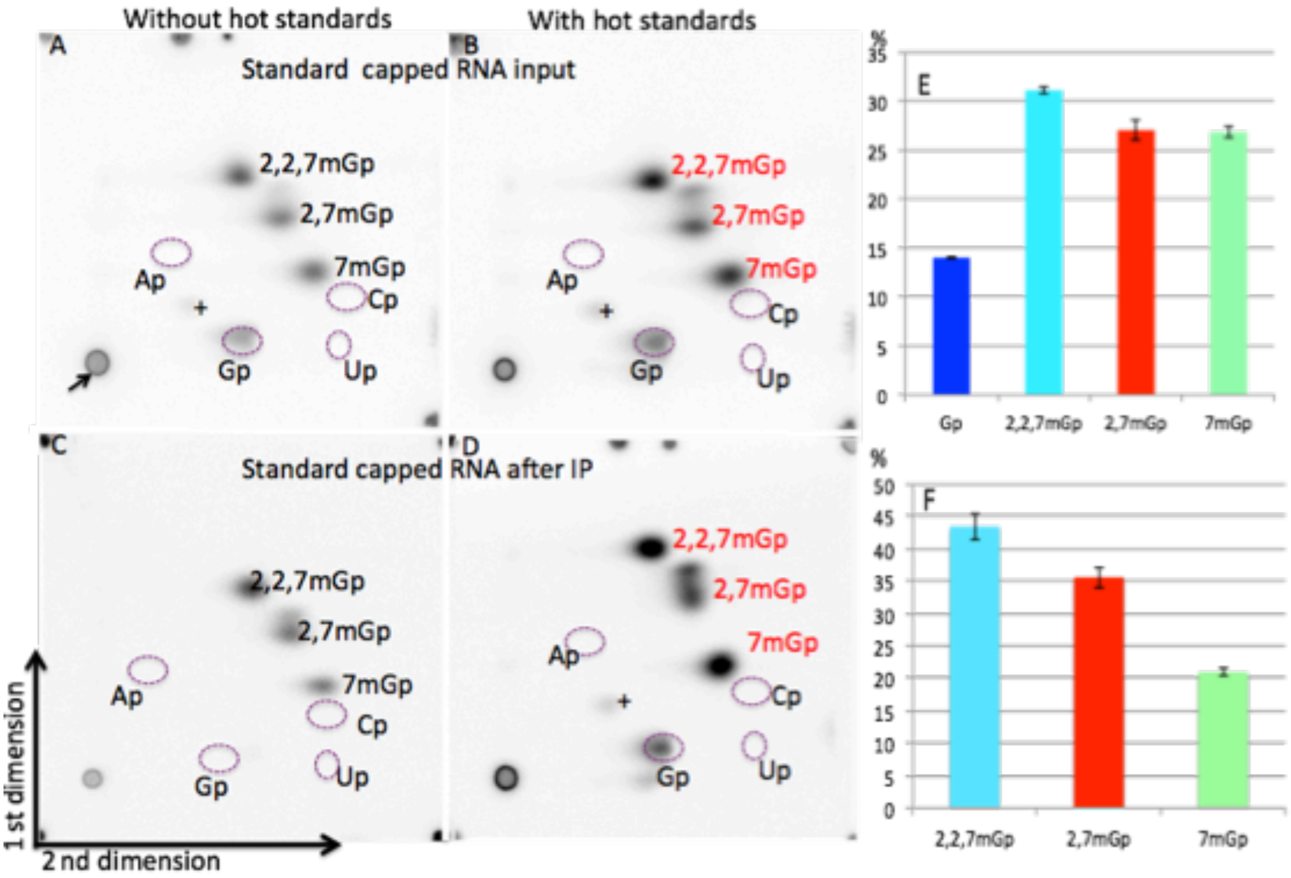

Figure S6: 2D-TLC replicates: The first replicate for each fraction is also displayed in Figure 4. See Figure 4's legend for details.

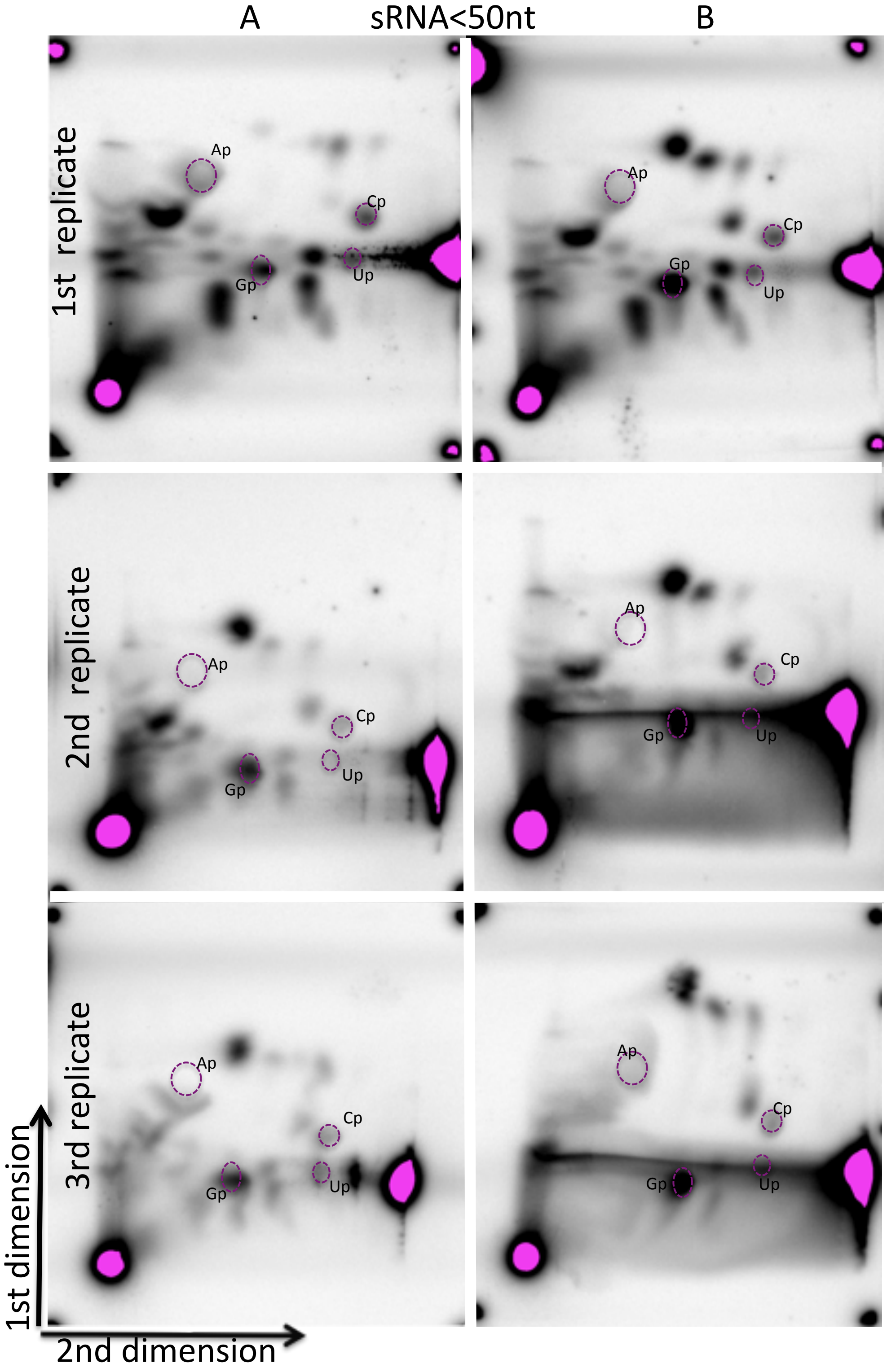

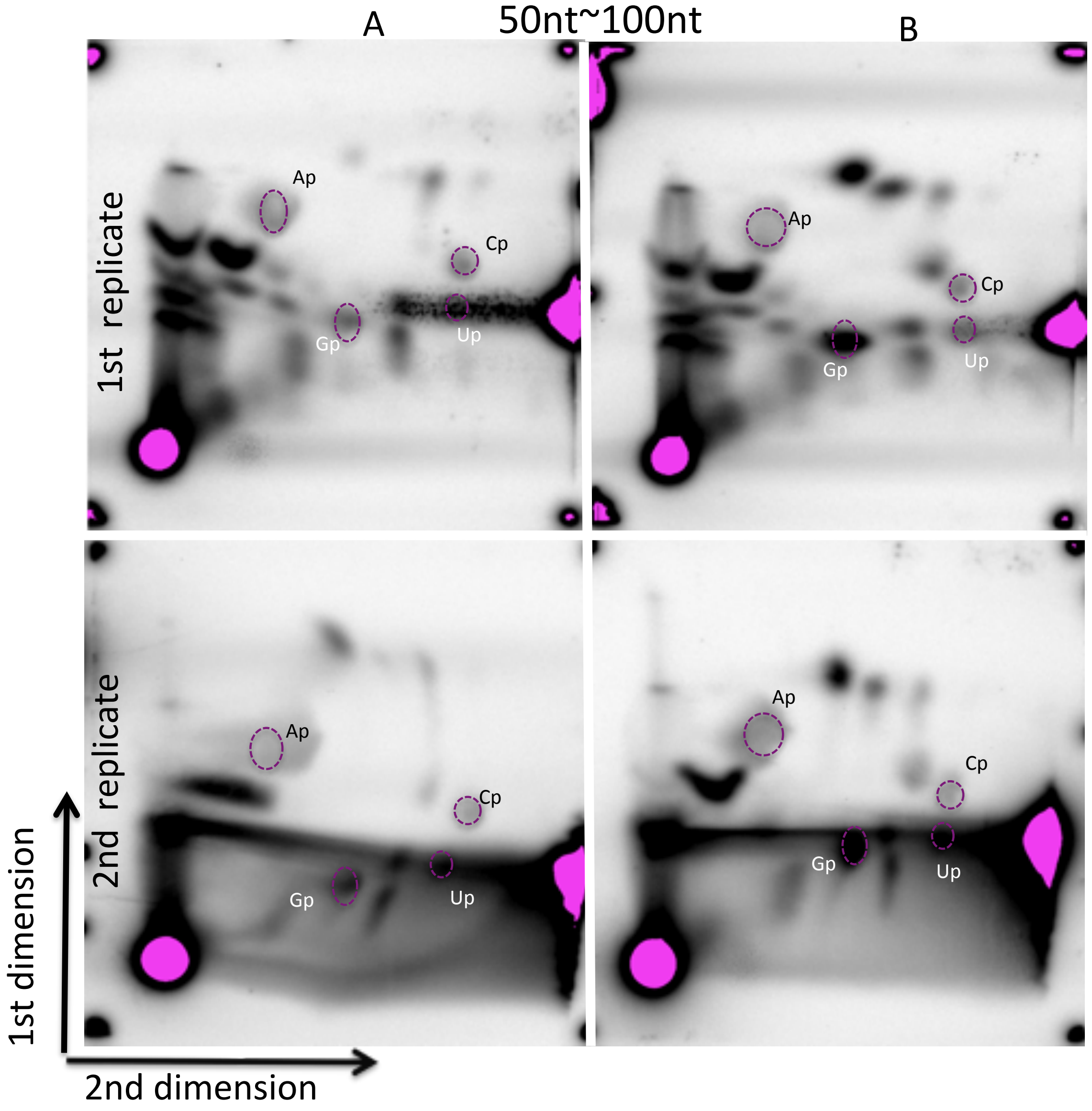

A
50nt~100nt
B
1st replicate
2nd replicate
Ap
Cp
Gp
Up
Ap
Cp
Gp
Up
Ap
Cp
Gp
Up
Ap
Cp
Gp
Up
1st dimension
2nd dimension

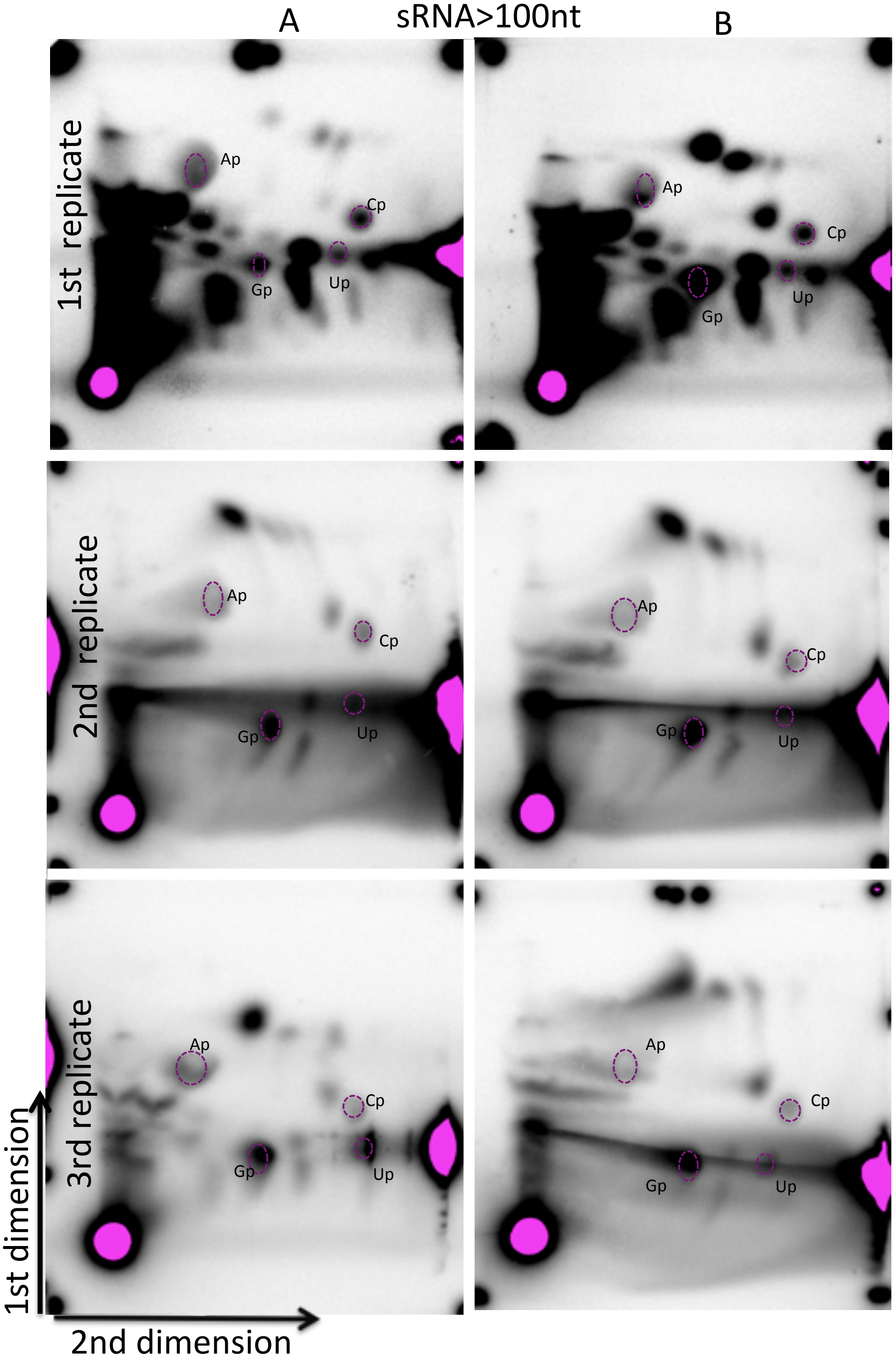

A
sRNA>100nt
B
1st replicate
2nd replicate
3rd replicate
Ap
Cp
Gp
Up
1st dimension
2nd dimension

Figure S7: Control experiment for TLC analysis. A: TAP and Antarctic Phosphatase omitted. B: RNA was treated with RNaseI prior to dephosphorylation with Antarctic phosphatase . C: TAP omitted.

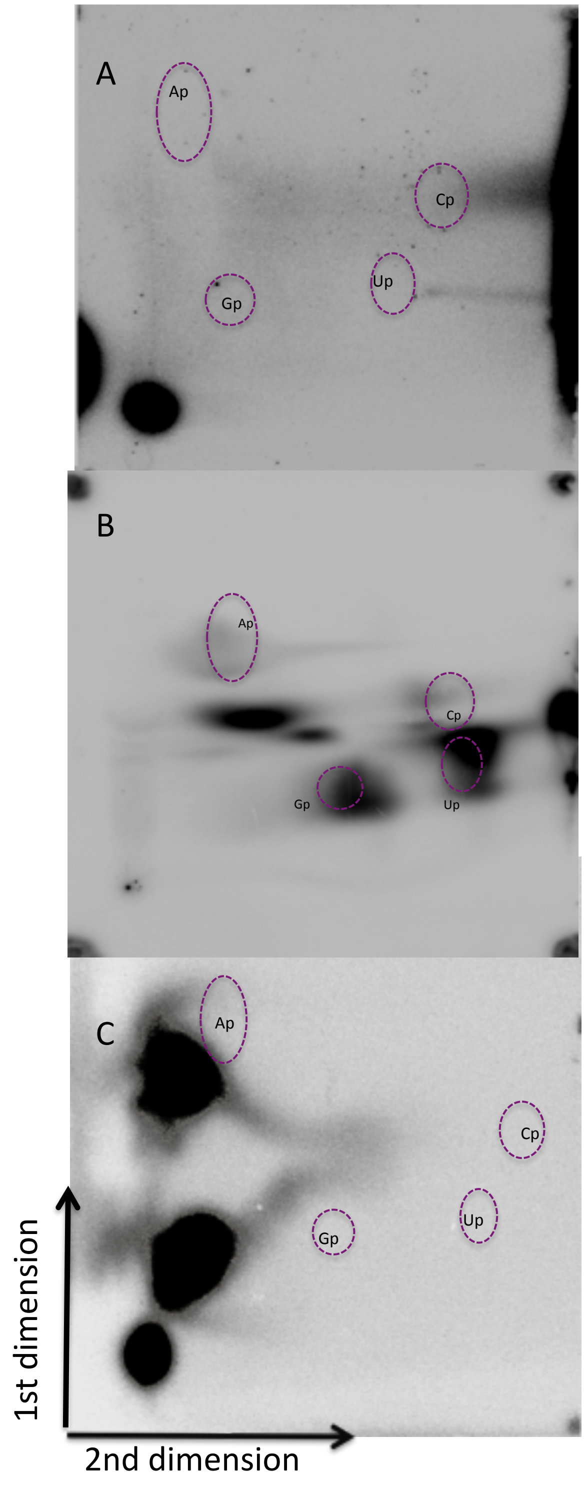

Figure S8: 2D-TLC analysis is for verification of the ability of T4 PNK to phosphorylate 5′ end of ribonucleosides. Labeling reaction without prior dephosphorylation in the presence of [γ-^{32}P]ATP (A) and ATP: [γ-^{32}P]ATP, 1:1 (B). Same reaction on ribomononucleotides dephosphorylated with ANT Antarctic phosphatase prior to labeling in presence of [γ-^{32}P]ATP (C) and ATP: [γ-^{32}P]ATP, 1:1 (D). The figure indicates two spots, X1 and X4, that could be 5′,3′-nucleotide diphoshphate.

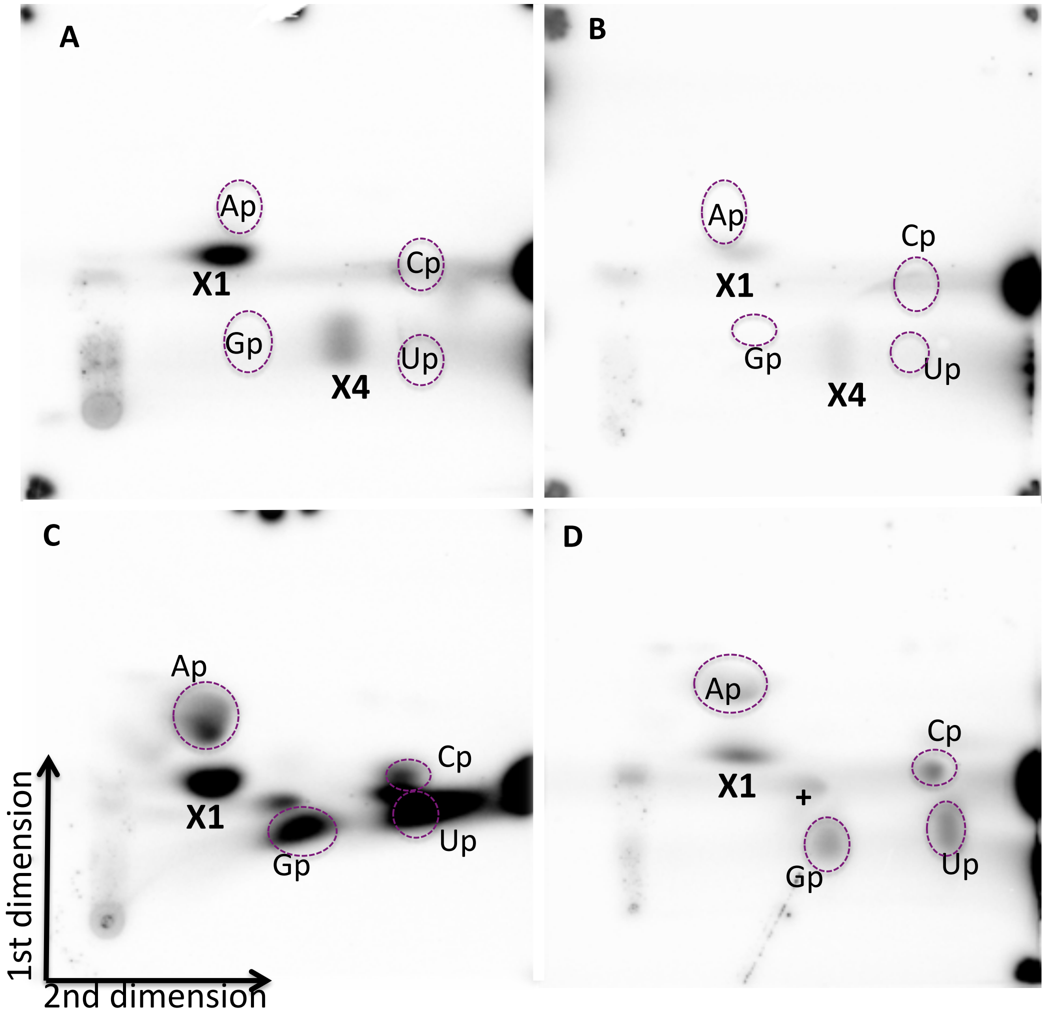

Figure S9: HPLC spectra of automated RNA fractionation using PLRP-300 column under unfolding condition at 60 °C, sample flow rate 50 µl / min. The gradient lasted for 80 min and started with 20 % B in A. Concentration of B buffer was raised to 31 % in 1 min, then to 41 % in 19 min, 70 % in 1 min, continue elution with 70 % 9 min. then wash column for 40 min with 20 % of buffer B. A 260 nm UV detector was used for detection.

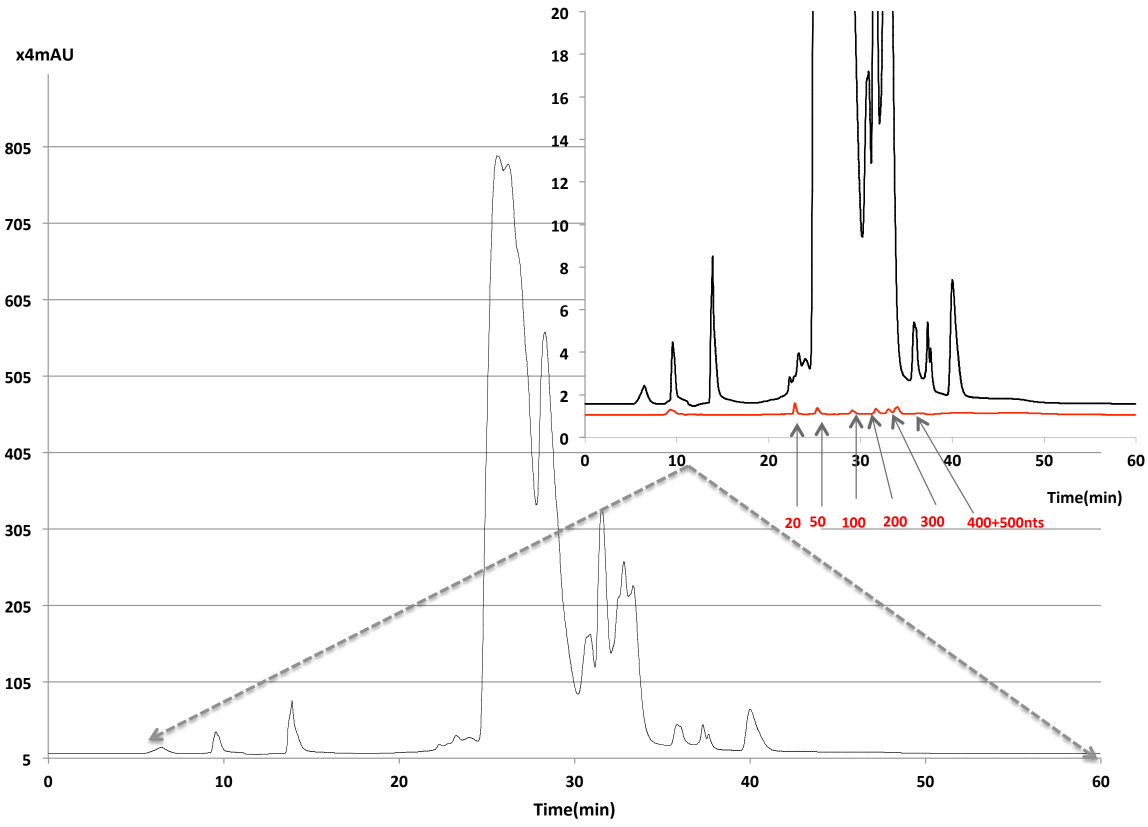

Figure S10: Distribution of the tags aligning to TSSs and TTS, defined as 200 bp windows centered at the start and end of GENCODE transcripts (excluding the known sRNAs). Expression levels are given in TPM.

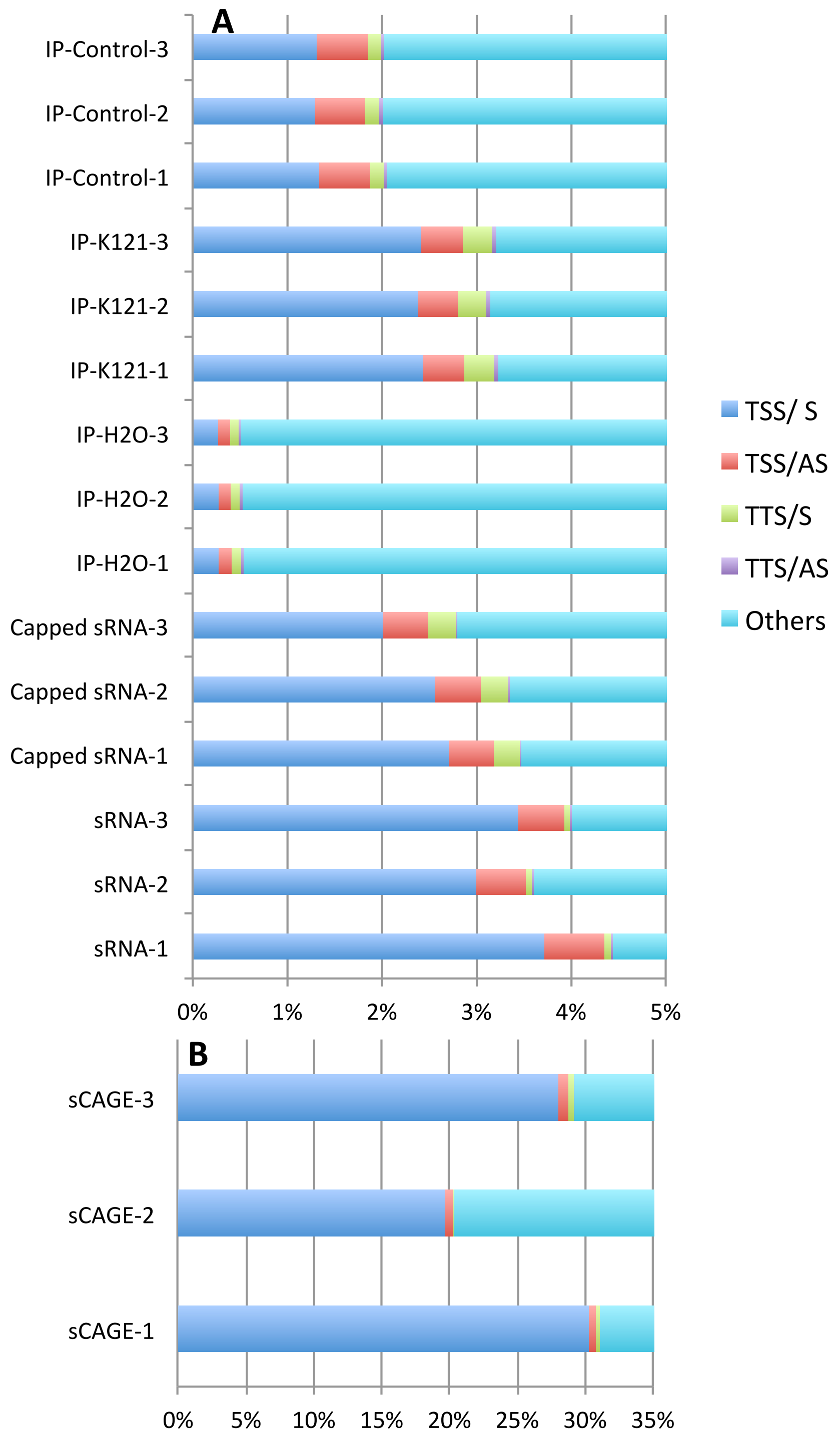

Figure S11: Chemical structure of modified ribonucleotide mentioned in this article according to Limbach et al. [34].

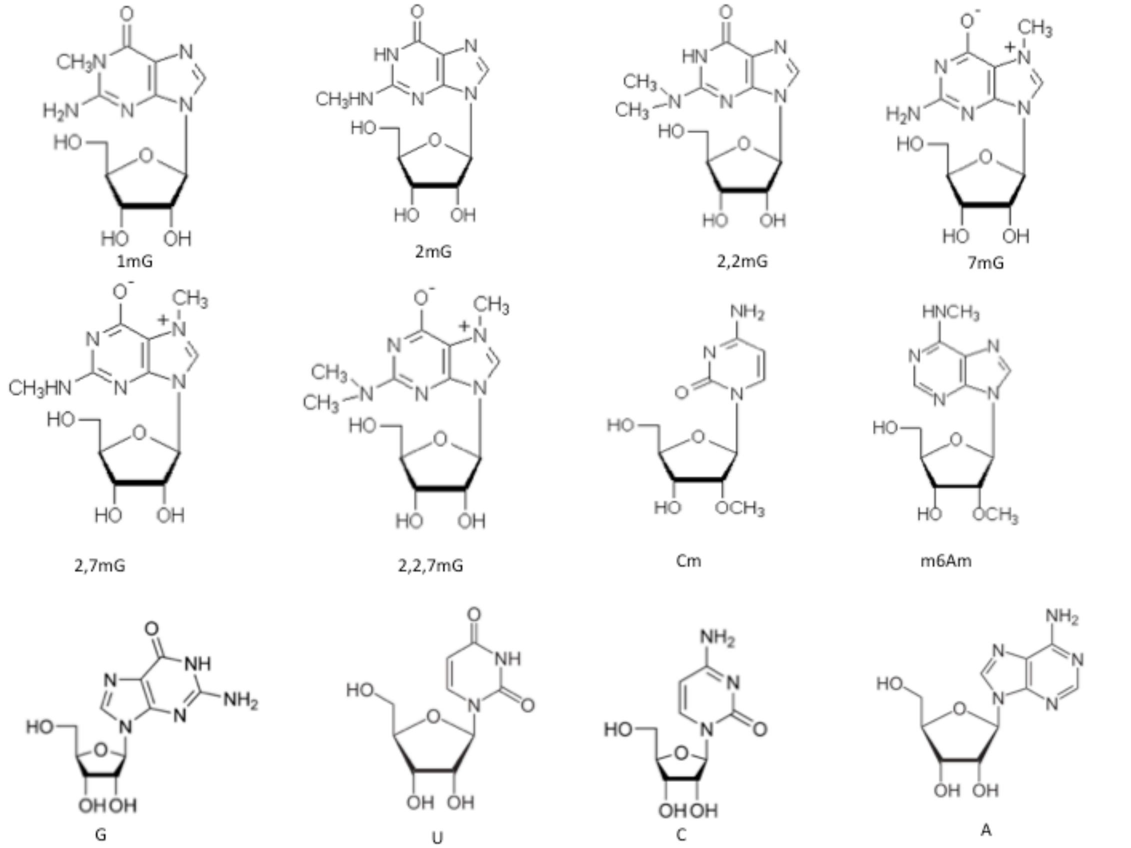

Supplement: File S1 — Contains the supplementary figures and their legends. (PDF) [file pone.0102895.s001.pdf]
